# Accurate Reconstruction of Cell and Particle Tracks from 3D Live Imaging Data

**DOI:** 10.1016/j.cels.2016.06.002

**Published:** 2016-07-27

**Authors:** Juliane Liepe, Aaron Sim, Helen Weavers, Laura Ward, Paul Martin, Michael P.H. Stumpf

**Affiliations:** 1 Department of Life Sciences, Imperial College London, London, SW7 2AZ, UK; 2 Centre for Integrative Systems Biology and Bioinformatics, Imperial College London, SW72AZ, UK; 3 School of Biochemistry, Biomedical Sciences, University of Bristol, Bristol, BS8 1TD, UK; 4 School of Physiology, Pharmacology and Neuroscience, Biomedical Sciences, University of Bristol, Bristol, BS8 1TD, UK; 5 School of Medicine, University of Cardiff, BS8 1TD, UK

## Abstract

Spatial structures often constrain the 3D movement of cells or particles in vivo, yet this information is obscured when microscopy data are analyzed using standard approaches. Here, we present methods, called unwrapping and Riemannian manifold learning, for mapping particle-tracking data along unseen and irregularly curved surfaces onto appropriate 2D representations. This is conceptually similar to the problem of reconstructing accurate geography from conventional Mercator maps, but our methods do not require prior knowledge of the environments’ physical structure. Unwrapping and Riemannian manifold learning accurately recover the underlying 2D geometry from 3D imaging data without the need for fiducial marks. They outperform standard x-y projections, and unlike standard dimensionality reduction techniques, they also successfully detect both bias and persistence in cell migration modes. We demonstrate these features on simulated data and zebrafish and *Drosophila* in vivo immune cell trajectory datasets. Software packages that implement unwrapping and Riemannian manifold learning are provided.

## Highlights

• Cell movement is often constrained, e.g., to surfaces of cellular structures
• We develop approaches to detect such constraints from in vivo live imaging data
• Accounting for these structures is necessary for correct analysis of cell tracks

## Graphical Abstract

**Figure:**
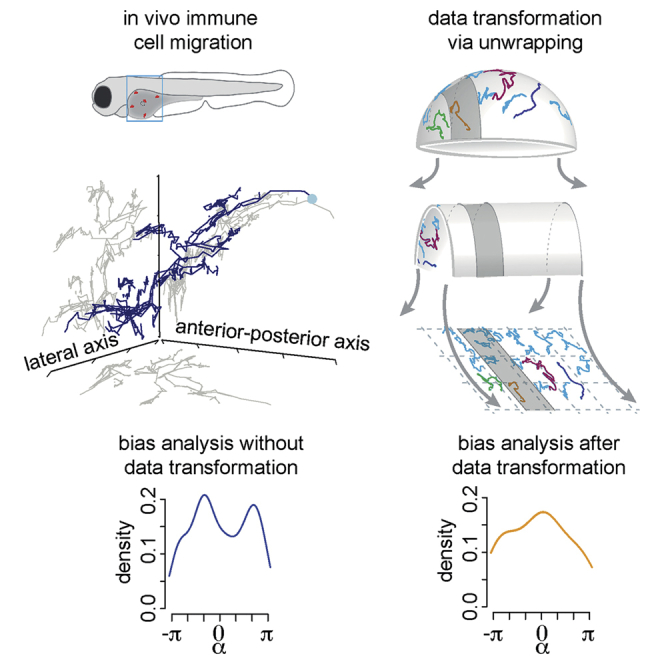

## Teaser

Liepe et al. present a set of tools that enable us to account for the spatial constraints acting on the motion of cells and particles in live imaging studies, and show how these allow us to accurately interpret these data.

## Introduction

The ability to image the often complex behavior of biological systems is indispensable to much of modern biological research. Developments such as fluorescence, high-resolution, and live-imaging techniques are now firmly established technologies in cellular and molecular biology (Megason and Fraser, 2007). The major advances in imaging include the move from 2D to 3D data acquisition, the transition from static images toward time-lapse movies and the ability to image objects in vivo in living animals rather than ex vivo studies of smaller systems (Arranz et al., 2014, Weigert et al., 2013). The study of cell migration is one notable beneficiary of these methodological developments. Together with new statistical and computational tools (Barbier de Reuille et al., 2015, Holmes et al., 2012, Jones et al., 2015), recent studies have already provided useful insights into many fundamental processes in immunology and developmental biology (Masopust and Schenkel, 2013, Phoon, 2006).

**Figure 1:**
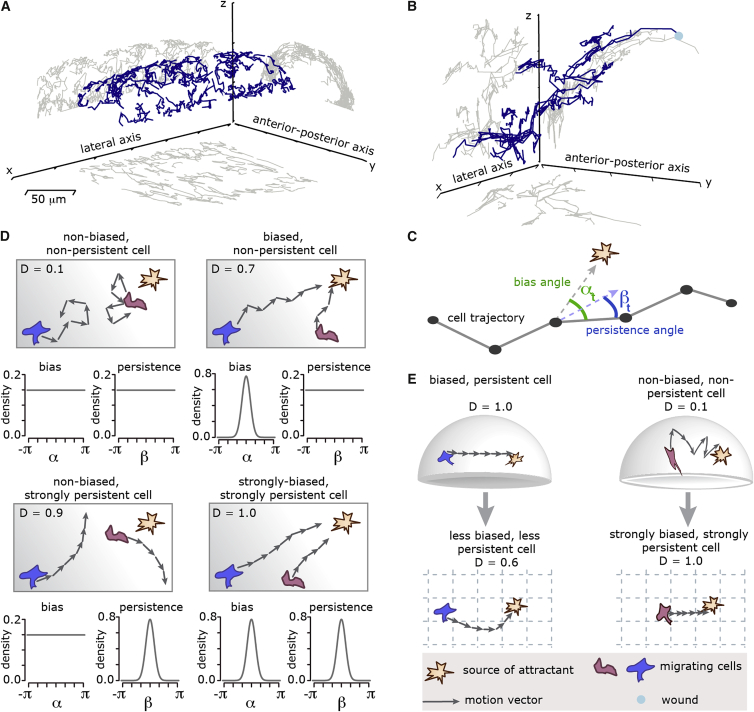
Directional Statistics of Cells Migrating on Curved Surfaces (A) 3D representation of haemocyte cell tracks extracted from *Drosophila* embryo (blue) with the xy-, xz- and yz-projections (gray). (B) 3D representation of neutrophil cell tracks extracted from laser wounded epidermis of the yolk syncytium of a zebrafish (blue) with the xy-, xz- and yz-projections (gray). Both, the datasets shown in (A and B) have a curvature, which is strong enough to induce analysis artifacts, but the same time weak enough to be analyzed using our proposed unwrapping method. (C) From each cell trajectory the indicated bias and persistence angles are measured for each time step. The bias angle describes the angle between a motion vector (a step of the cell) and the direction pointing toward the attractant. The persistence angle describes the angle between two consecutive motion vectors. All the measured bias and persistence angles of each cell track built the bias and persistence distributions, from which the strength of bias and persistence can be estimated. (D) Four types of random walks are sketched as cartoons, visualizing bias and persistence. For these random walk the expected bias and persistence distributions can be obtained mathematically and are here plotted as an example. The straightness index (D) is noted as a reference (see supplemental information for the definition of the straightness index). (E) Artifacts that appear when random walks happen on curved surfaces but are analyzed in the 2D projections.

Movements captured in 3D are, however, rarely unconstrained 3D motions. They often take place in 1D (along e.g., blood vessels, microtubules, or actin filaments) or on 2D surfaces (e.g., curved cell walls or the interstitial medium in layered tissues such as the epithelium). Ignoring these structures during analysis can produce results that are skewed and erroneous. (Figure 1). Even when acknowledged, these lower-dimensional spaces can be highly curved and irregularly shaped. For example when a cell or molecule moves along a curved surface (Figure 1E, top), standard 2D projections, including e.g., principal component analysis (PCA), can introduce curvature into its track where there is none (Figure 1E, bottom left) or artificially smooth a track (Figure 1E, bottom right).

It is therefore important to acknowledge underlying lower-dimensional structures when analyzing random walks; however, these underlying structures are rarely an ideal curved surface. These lower-dimensional spaces can be highly curved and irregularly shaped. In such cases, the commonly used linear dimensional reduction methods such as principal component analysis (PCA) are no longer appropriate for either data visualization or data analysis. Here, we present two methods for identifying a 2D coordinate representation of a given 3D point-cloud dataset that preserves the geometrical information of its hidden embedded surfaces. Both methods are non-linear generalizations of both linear projections onto pre-defined 2D planes and PCA (Jolliffe, 2013). As shown below for migrating cells on curved surfaces, the methods are able to detect the bias and persistence modes of biological random walks models. The first approach, which we refer to as “unwrapping,” is an intuitive two-step process that is particularly applicable to scenarios where the underlying 2D surfaces have relatively simple structures—specifically, convex surfaces with zero or small intrinsic curvatures (e.g., local patches on cylindrical or ellipsoid-like manifolds). This prior knowledge of the surface geometry allows the method to be effective even when the data are relatively sparse. The second method, “Riemannian manifold learning,” is an adaptation of an existing method in machine learning that, while somewhat more abstract, can be applied to surfaces that are irregularly shaped, non-convex, or highly curved without prior knowledge of underlying structures. We provide a set of example code for both methods in supplemental information, including pure R scripts as well as Jupyter notebooks coded in R and Python (Data S1 and Data S2).

## Results

We provide a brief description of the two methods and demonstrate their application by extracting quantitative information from a set of simulated and experimental data of cell migration on complex surfaces.

### Method 1: Unwrapping

**Figure 2:**
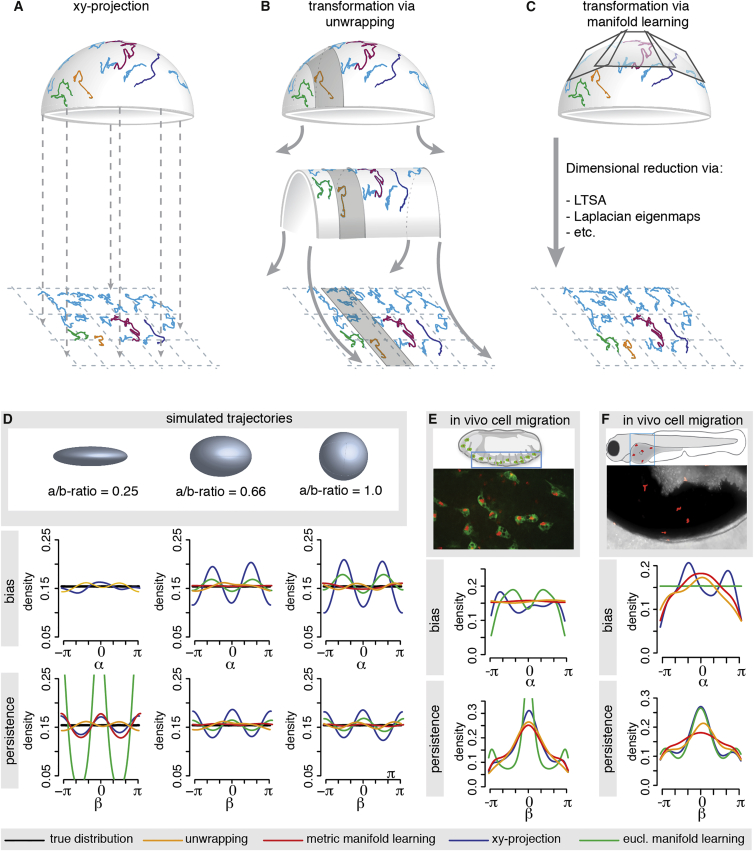
Methods for Manifold Learning and Applications (A–C) Shown are example trajectories on a hemi-sphere and their transformation via one of the discussed methods. The 3D cell tracks are simply projected onto the xy-plane (A). The 3D cell tracks are transformed via unwrapping (B) or via well-known manifold learning methods (e.g., LTSA) (C). (D) Random walk trajectories (in absence of any bias or persistence) are simulated on the displayed curved surfaces and then transformed with xy-projection, unwrapping, Euclidian manifold learning and Riemannian, or metric, manifold learning, respectively. The resulting bias and persistence distributions are compared with the respective true distribution (black), which are uniform for this random walk model. For the ellipsoid with the most extreme aspect ratio (i.e., the “thinnest” shape), the manifold learning approach was unsuccessful as it incorrectly interpreted the data as belonging to a 1D line. In this case, there was insufficient data to correctly reveal the spatial extent of one dimension. (E) Application of the unwrapping method and manifold learning methods to haemocyte cell tracks extracted from a *D. melanogaster* embryo and their comparison to the xy-projection. Shown is a schematic of the embryo and a snapshot from the video microscopy imaging. Haemocytes (green) were tracked via their nucleus (red). (F) Application of the unwrapping method and manifold learning methods to neutrophil cell tracks extracted from the epidermis overlying the yolk syncytium of a zebrafish and their comparison to the xy-projection. The epidermis was wounded with a laser before image acquisition. Shown is a schematic of the zebrafish with the imaged area and a snapshot from the video microscopy imaging with the neutrophils in red.

In a standard xy projection, information about the cell’s position (illustrated here along a convex, curved 2D surface) in the z dimension is simply ignored. By contrast, our unwrapping method maps the points on the curved 2D surface to appropriate coordinates by fitting a set of ellipses to a succession of data slices (indicated in gray in Figure 2B), and then “unrolls” these 1D strips onto straight lines. The resulting flattened representation of the data can then be analyzed using conventional tools for cell migration analysis. The method proceeds in two steps. First, unwrapping projects data that lie on part of a convex surface onto a cylindrical-like surface, i.e., flat in one direction (Figure 2B, see also Supplemental Information). In the second step, the process is repeated in the orthogonal dimension and the data are mapped from the intrinsically flat cylindrical surface onto a flat plane.

### Method 2: Riemannian Manifold Learning

All 2D coordinate maps of a given curved surface will misrepresent the latter’s geometry to some extent (consider, for example, the inflated sizes of countries near the polar regions in the Mercator projection of the world map). “Riemannian manifold learning” (or equivalently, metric manifold learning) is a method that allows one to quantify this misrepresentation for any given 2D coordinate map, and to then use this geometric information in any subsequent analysis of the data (Figure 2C). The method was first introduced as the LEARNMETRIC algorithm in Perraul-Joncas and Meila (2013) and is a straightforward adaptation of the non-linear dimensional reduction techniques common in the field of statistical machine learning.

The working principle behind this method is that the geometry of any embedded 2D surface is entirely encoded in a position-dependent 2 × 2 matrix known as the “metric tensor.” This metric can be consistently inferred, without using prior knowledge or making assumptions about the geometry, from the set of 3D data points and its corresponding set of 2D coordinate maps. In all the examples in this paper, we obtain the 2D coordinates using the locally linear embedding method. Nevertheless, as discussed in Perraul-Joncas and Meila (2013), the method is applicable to any other smooth, invertible map such as ISOMAP, Laplacian Eigenmaps, or even the unwrapping method introduced above. We have included a brief introduction of the relevant mathematical details in the supplemental information.

Given this 2D coordinate representation of the data, one then incorporates the geometrical information from the metric when calculating the usual statistics of interest that describe cell biological data, such as turning angles, step-lengths, and cell velocities. We note that despite being generally applicable to any open surface, this method requires more detailed input by the user than the unwrapping method. Specifically, this is the so-called bandwidth parameter intrinsic to manifold-learning algorithms; this is effectively the extent to which the neighborhood of a point can be considered to be a flat surface (see Supplemental Information for details).

### Unwrapping Recovers Random Walk Characteristics

To test and characterize our approach, we validate the two methods on a set of simulated (in silico) datasets, before applying it to data obtained by fluorescent time-lapse microscopy imaging.

To start, we simulated cell tracks based on a Brownian motion type (non-biased and non-persistent, as described in Figures 1C and 1D) random walk model on several surfaces of varying curvatures, ranging from a thinly-stretched ellipsoid to a sphere (details of the random walk models are described in the Supplemental Experimental Procedures). This type of random walk necessarily produces flat angular distribution (Figure 2D, “true distribution”), which we compare to the computed angular distributions based on the simple the xy-projection, the unwrapping method, and the manifold learning method (with and without incorporating geometrical information) (Figure 2D). We observe the largest deviation from the true angular distribution for the oft-employed xy-projection, highlighting the need for data transformation methods, especially for estimates of the bias distribution on more highly curved surfaces. Both the unwrapping and the metric manifold learning methods manage to recover the true distribution with only small deviations. The more commonly employed manifold learning approach that omits the metric (i.e., “Euclidian*”* manifold learning) performs significantly worse than the metric manifold learning method, especially on very narrow ellipsoids.

To demonstrate that unwrapping and metric manifold learning methods are generalizable, we tested them on in total six different geometries and simulated data obtained from three different random walk models (Figures S1A–S1C, related to Figure 2). The unwrapping method recovers all bias angle distributions and shows improvements for the persistence angle distributions compared to conventional xy-projections. The performance of the metric manifold learning method is slightly better still than the unwrapping method. To quantify the performance of the different methods, we computed the deviation distance of the angle distributions obtained through each of the methods from the true angle distributions (Figure S2, related to Figure 2). The unwrapping method and the metric manifold learning method perform better than the simple xy-projection on all tested surfaces. This analysis demonstrates that, in principle, both the unwrapping and the metric manifold learning algorithm are well-suited methods for the analysis of cell migration on curved surfaces. Given suitable high-resolution data they can also be applied to study intra-cellular movement of e.g., proteins on cellular structures such as the endoplasmic reticulum or the mitochondria.

### Unwrapping Detects Biased-Persistent Immune Cell Migration

Next, we analyzed bias and persistence in the migratory behavior of immune cells in vivo. Specifically, we observed haemocyte migration in the embryo of the fruit fly *Drosophila* and neutrophil migration in the epidermis overlying the yolk syncytium of a zebrafish in response to wounding. We extracted the data from 3D time-lapse fluorescent movies and track the cells over time (for details see Supplemental Information). The haemocytes migrated in a constrained, pseudo-2D region beneath the surface of the embryo and did not enter deeper tissue layers at this developmental stage (Figure 1A, Movie S1, and Movie S3). This is consistent with previous observations, which report that haemocytes have no spatial bias toward any particular point and move in non-biased, non-persistent manner (Davis et al., 2012). Accordingly, we observe a non-uniform persistence distribution when haemocyte motion is analyzed with the unwrapping or metric manifold learning methods (Figure 2E). Analysis of the xy-projection results in artifacts, which indicates a bias toward an arbitrary point and overestimates the strength of the persistence. Additional transformations of the data highlight the deviations between the unwrapping method, the metric manifold learning method, and the xy-projection (Figure S3, related to Figure 2).

We also analyzed the response of neutrophils to a wound (Figure S2F, related to Figure 2). From previous studies (Holmes et al., 2012, Taylor et al., 2013), we know that neutrophils constitute the first line of defense and directly migrate persistently toward wounds, i.e., they show biased persistent motion. The cells in this example migrate on a curved surface constrained by the epidermis overlying the yolk syncytium (Figure 1B, Movie S2, and Movie S4). Unwrapping the data and analyzing the resulting distributions shows a clear bias of the neutrophils toward the wound with some level of persistence, both of which are confirmed by the metric manifold learning method. By contrast, analysis of the xy-projection results in strong artifacts for bias and persistence, missing the bias entirely, which is not biologically reasonable given the neutrophils’ function. Further, when we use PCA to reduce the dimensionality, as is common in many applications where one would like to visualize a 2D representation of a higher-dimensional dataset, the resulting angular distributions show even more pronounced artifacts than the simple xy-projection (Figures S1F and S1G). These results highlight the need for manifold learning techniques that go beyond simple linear projections; but they also show that our rather intuitive and data-driven unwrapping approach can provide an adequate representation of the experimental data.

## Discussion

It is well-known that image processing techniques can introduce artifacts into cell migration analysis (Beltman et al., 2009). The problem highlighted and tackled here is more closely related to finding the right representation of data (and has obvious parallels with cartographic projections). In the two examples of in vivo data analysis, we have shown that an appropriate metric manifold learning method is required to detect a well-established biological behavior: the bias of the zebrafish neutrophils toward a wound. Without these methods, we would have wrongly concluded that *Drosophila* haemocytes migrate with a bias in absence of an obvious attractant source based only on the xy-projection. Erroneous or incorrect analysis has implications beyond wrong conclusions (Sim et al., 2015): for example, poor analysis can render valuable patient and animal data useless.

Given the importance of constrained cellular and molecular movement throughout cellular and developmental biology and medicine, unwrapping and metric manifold learning methods will be broadly applicable. The development of quasi-conformal mapping methods (Appleboim et al., 2006) has been driven largely by the needs of the medical imaging community for 2D image representations of human organs with minimal geometric distortion (Schwartz and Merker, 1986). These methods, however, presume the ability to either capture a high-resolution image of the surface or to construct a triangulated mesh covering. In many contemporary biological applications these surfaces are rarely imaged directly with their existence only inferred indirectly from the migration tracks of the imaged objects. Obtaining an image of the surface would often require additional in vivo staining or generation of suitable tissue markers, both of which carries the risk of interfering with the image acquisition of the actual target cells. Both unwrapping and metric manifold learning relieve this need, as they require no fiducial marks or characterized in situ spatial constraints.

## Experimental Procedures

### Data Acquisition

*Drosophila* were maintained on cornmeal agar fly food, supplemented with dried yeast, and handled according to standard protocols (Greenspan, 2004). Stage 15 embryos were collected from overnight apple juice plates at 25°C (ubi-EcadherinGFP, serpent-Gal4 > UAS-GFP;UAS-redstinger), carefully dechorionated in 50% bleach, washed thoroughly with distilled water and mounted on a glass slide in a drop of 10S voltalef oil (VWR). Movies were collected at 30 s/frame on a PerkinElmer UltraView spinning disc microscope using a ×40 oil immersion lens.

5-days-post-fertilization Tg(Lyz:dsRed)nz zebrafish larvae (Hall et al., 2007) were mounted laterally in 1.5% low-melting agarose (Sigma) in a glass-bottomed petri dish containing Danieau's solution and 0.01 mg/ml MS-222 (Sigma). The epidermis overlying the yolk syncytium was wounded using a UV-nitrogen laser (Coumarin 440 nm dye cell) coupled to a Zeiss Axioplan 2 microscope (Micropoint Laser System, Photonic Instruments) with a 40× water immersion objective. Movies were collected at 1 min/frame using a Leica SP5-II AOBS confocal laser scanning microscope attached to a Leica DM I6000 inverted microscope with a ×20 glycerol lens.

Further methods and any associated references are available in the Supplemental Information.

## Supplemental Information

Document S1. Supplemental Experimental Procedures and Figures S1-S3

Movie S1. Shown Are Example Raw Data for the Unwounded *Drosophila* Embryo Data Set Analyzed in Figures 1A, 2E, and S1, Related to Figures 1 and 2 Time-lapse movie of the dynamic behavior of Drosophila immune cells (heamocytes) in unwounded tissue. Epithelial cells are labeled using E-cadherin-GFP (green cell outlines), immune cell nuclei are labeled using nuclear Red-Stinger (red) and immune cell cytoplasm using cytoplasmic GFP (green) both driven by srp-Gal4.

Movie S2. Shown Are Example Raw Data for the Wounded Zebrafish Data Set Analyzed in Figures 1B and 2F, Related to Figures 1 and 2 Time-lapse movie of the dynamic behavior of zebrafish immune cells (neutrophils) in laser-induced wounded tissue. Immune cells are labeled using cytoplasmic dsRed (red) driven by the lysozyme C (lyz) promoter.

Movie S3. Shown Are the Haemocyte Cell Tracks Extracted from *Drosophila* Embryos in 3D, Related to Figures 1 and 2 The different rotation angles show the curvature of the space the haemocytes are migrating in.

Movie S4. Shown Are the Neutrophil Cell Tracks Extracted from Zebrafish Embryo, Related to Figures 1 and 2 The epidermis overlying the yolk syncytium was wounded. The location of the wound is indicated by a light blue dot.

Data S1. This Folder Contains the Two Jupyter Notebooks for Unwrapping and Manifold Learning, Related to Experimental Procedures Furthermore it includes the two websites for both methods. Example data are stored in the folder “SimulationData.”

Data S2. This Folder Contains All Described R Scripts and the Provided Example Data in Order to Perform Unwrapping on Simulated and In Vivo Data, Related to Experimental Procedures

Document S2. Article plus Supplemental Information
