# Supplementary material for: Accurate Reconstruction of Cell and Particle Tracks from 3D Live Imaging Data
Source: Cell Syst. 2016 Jul 27;3(1):102–7. doi: 10.1016/j.cels.2016.06.002 (PMC4963212; doi:10.1016/j.cels.2016.06.002)
Supplement: Document S1. Supplemental Experimental Procedures and Figures S1-S3 [file mmc1.pdf]

Cell Systems, Volume *3*

Supplemental Information

Accurate Reconstruction of Cell

and Particle Tracks from 3D Live Imaging Data

Juliane Liepe, Aaron Sim, Helen Weavers, Laura Ward, Paul Martin, and Michael P.H. Stumpf

__Accurate reconstruction of cell and particle tracks from 3D live imaging data__

Juliane Liepe[*1;2], Aaron Sim[*1;2], Helen Weavers[3], Laura Ward[4], Paul Martin[3;4;5], Michael PH Stumpf[1;2]

[1]Department of Life Sciences, Imperial College London, London, UK, SW7 2AZ
[2]Centre for Integrative Systems Biology and Bioinformatics, Imperial College London, UK, SW72AZ
[3]Department of Biochemistry, Medical Sciences, University of Bristol, Bristol, UK, BS8 1TD
[4]School of Physiology and Pharmacology, University of Bristol, UK, BS8 1TD
[5]School of Medicine, University of Cardiff, UK, BS8 1TD

* These authors equally contributed to the work.

1. Supplemental Experimental Procedures

1.1. Tutorial: How to unwrap cell trajectory data using Jupyter

In this section we provide a brief example how to unwrap trajectory data that lie on a curved surface. The required code is provided in Suppl. Materials as an html file as well as a Jupyter notebook. You can follow step by step the next sections in parallel with the Jupyter notebook.

Prerequisites.
The code is written in R. To run the Jupyter notebook you need: (i) an installation of the Jupyter notebook (http://Jupyter.org/), (ii) an installation of the R statistical environment (http://www.r-project.org/) and (iii) the R Kernel for the Jupyter notebook, which can be installed from https://github.com/IRkernel/IRkernel. For the latter the instructions found at http://www.michaelpacer.com/maths/r-kernel-for-ipython-notebook are good for installing this under OSX. Visualizing the output in R requires the *rgl* package, which can be installed using your
R environment. Providing that these packages are in place the code in this notebook should run without any further problems.

Installation.
The Jupyter notebook (previously *IPython notebook*) requires a working installation of Python in the first place; most Python distributions aimed at scientific computing contain the relevant files and packages. The *Anaconda* (*https://www.continuum.io/downloads*) is, in our experience, particularly straightforward to install, use, and maintain. Installation can be done via the provided installers (for Windows, OSX and Linux), or from the command line (the website https://www.continuum.io/downloads contains instructions for the various versions).

Maintaining and upgrading the distribution's packages is done using the *conda* package-manager. To upgrade the jupyter notebook, for example, at the command line write

```
> conda upgrade jupyter
```

This installs all the files required for using *Jupyter* in conjunction with Python. Other kernels can be installed as described on the relevant webpages, which are linked to at https://github.com/ipython/ipython/wiki/IPython-kernels-for-other-languages. For the *R* kernel the *conda* distribution offers a convenient way of installing the relevant packages (assuming that a recent *R* installation is present),

```
> conda install –c r r-essentials
```

(see https://www.continuum.io/blog/developer/jupyter-and-conda-r for further details).

How to execute the Jupyter notebook.
The Jupyter notebooks are available in the folder *Jupyter*. A step-by-step guide is presented as a web page called *UnwRapping .html (Data S1)*. To execute the notebook, at the command line enter (in the Jupyter folder Data S1)

```
> jupyter notebook
```

This will start the default browser with and the loads the contents of the directory,

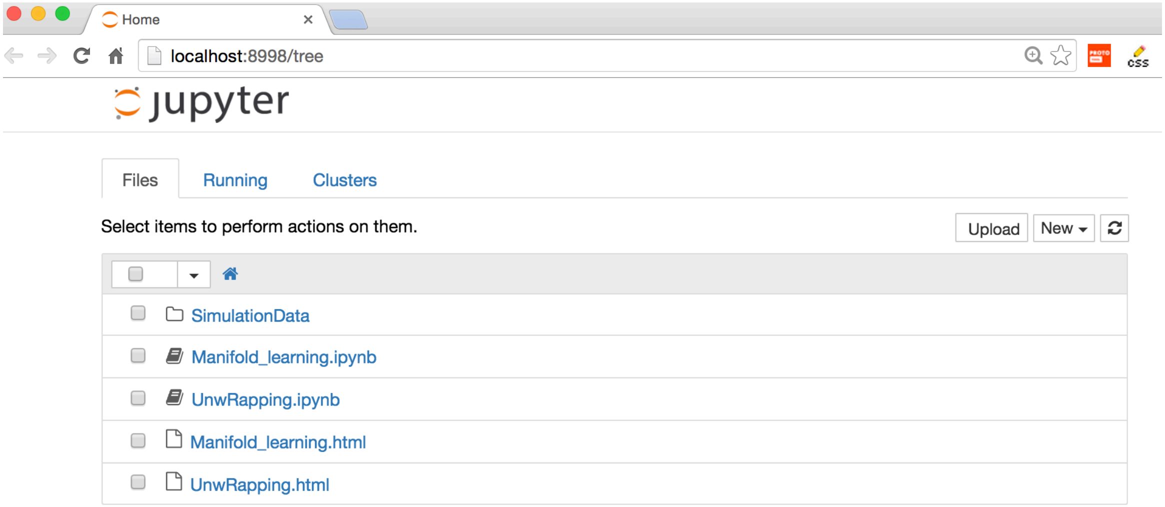

Clicking on the relevant Jupyer notebook (the files with an extension ".ipynb") will then start the relevant Jupyter notebook.

Running *Mannifold_learning.ipynb* will be straightforward with any recent Python installation; running the *UnwRapping.ipynb* notebook will require the installation of the *R* kernel.

Routines for Unwrapping Data. We first define a set of necessary routines. *getAngleBias()* lets us define the angle to the wound (target). *getAnglePersistence()* determines the angle between successive steps and hence measures the persistence. *transformData()* and *unwrapData3D()* are the routines that transform the data and unwrap them into a flat space. The code is provided in the Jupyter notebook.

Data preparation. We provide an example data set in Suppl. Materials (*exampleDataBPRW.csv*) in the folder *SimulatedData*. This data set describes simulated cell trajectories based on a biased persistent random walk on an ellipsoid surface. The data are saved as *csv* format, which can be opened in any text editor or Excel. The data file has to be provided in a specific layout: It should contain 4 columns, where the first column is the cell track ID (id), and the second, third and fourth columns are the x-, y- and z-coordinates of the cell tracks, respectively. The rows are then the individual time points for all cell tracks. The user can replace this data file with own data files. We use the same data format for all methods provided.

The target of the biased cells (wound) is at position (-7; 0; 0), which needs to be defined in the first step. Next the data are imported and reformatted (point 6 in the notebook).

Plot Data in 3D. We begin by plotting the trajectory data in 3D (using *rgl*). This allows us to get an idea of the geometry of the data (see notebook point 6). We can then clearly see that the trajectory data lie on a surface of an ellipsoid with radii 6; 6 and 18.

Analysis of random walk statistics in the 2D projection. Before unwrapping the data we calculate the bias and persistence of the random walk data in the conventional projection down to 2D, *i.e.* using the *x*- and *y*-coordinates only. We call the routines *getAngleBias()* and *getAnglePersistence()* by passing the relevant coordinates.

Unwrapping of the data onto a flat manifold. We next transform the data onto a flat space using the unwrapping method by calling the routine *unwrapData()*. If a simple representation of the manifold is available (such as a cylinder or ellipsoid) then we can unwrap the data by mapping the correct positions on the manifold (akin to cartographic projections) in a way that maintains the angles correctly. The routine will create a 3D graphical presentation of the progress of the unwrapping procedure. Firstly, the original data are plotted in 3D and then shifted to be suitable for the unwrapping using the routine *transformData().* The original data are then clustered along the *x*-axis and plotted in 3D, where each cluster is shown in a different color. Next, an ellipse is fitted to each cluster. The data are then unrolled onto a new space based on the characteristics of the fitted ellipse (for exact details see section 4). This first step is an approximation to manifold learning techniques. In the following the data obtained from the first step are further unrolled by fitting an ellipse onto a flat space. The routine will plot the transformed data in grey.

Analysis of random walk statistics in the manifold projection. We can now calculate the bias and persistence of the random walk data in the 2D manifold projection. Again, this is done via the routines *getAngleBias()* and *getAnglePersistence()* by passing the relevant coordinates.

Comparison of inferred biased and persistence behavior in the 2D x-y and unwrapped (manifold) projection. Finally we can compare the computed statistics for the *xy*-projection with the statistics computed from the unwrapped data. This is for example done via plotting the histograms and densities for the bias and persistent distributions. Doing so, we observe strong artifacts in the obtained distributions based on the *xy*-projection. On the contrary, unwrapping manages to recover the expected bias and persistence distributions.

1.2. Tutorial: How to unwrap cell trajectory data using R

Additionally to the Jupyter notebook we also provide the plain R code for unwrapping data that lie on a curved surface. The equivalent routine for the above-described example of a biased-persistent random walk can be found in the folder *exampleCode_Unwrapping_1_BPRW* (part of Data S2). Furthermore we provide the same routine for a purely persistent random walk (without bias) in the folder *exampleCode_Unwrapping_2_PRW* (part of Data S2) and for an *in vivo* data set extracted from a fly embryo in the folder *exampleCode_Unwrapping_3_inVivo* (part of Data S2). In the latter data set the fly was wounded with a laser. In this example we find that without unwrapping it is possible to detect a weak bias towards the wound. However, after unwrapping the data it becomes apparent that the cells are strongly biased towards the wound but also into the opposite direction, *i.e*. away from the wound.

Installation and Prerequisites.
You need to install the R statistical environment (http://www.r-project.org/). You can download the precompiled binary file for installation for most computer platforms (http://cran.ma.imperial.ac.uk/). Simply download the binary file suitable for your platform, double click it to start installation. More advanced user might chose to install R from source code.

For visualization purpose you need the R library 'rgl'. Again, you can download the binary file for installation from https://cran.r-project.org/web/packages/rgl/index.html. Alternatively, open a terminal, start R by typing
R
followed by enter. Then type

```
install.packages('rgl')
```

which will also initiate the installation of the library.

How to use the R scripts.
First of all open a terminal. On most Macs you can find the terminal in the folder 'Applications/Utilities'. When the terminal is started, it usually links to your home directory. Type 'pwd' to know in which directory you currently are. Change the directory to one of the 3 example code folders by typing in the terminal for example:

```
cd WorkFolder/exampleCode_Unwrapping/ exampleCode_Unwrapping_1_BPRW
```

If you are new to terminal and the related commands, please refer to http://ss64.com/osx/

Then start R by typing in the terminal
`R`
followed by enter.
To run the example script type
`source("runAnalysis.r")`

The script will first read in the data 'exampleDataBPRW.csv' located in the folder 'simulatedData'. The data are saved as *csv* format, which can be opened in any text editor or Excel. The data file has to be provided in a specific layout: It should contain 4 columns, where the first column is the cell track ID (id), and the second, third and fourth columns are the x-, y- and z-coordinates of the cell tracks, respectively. The rows are then the individual time points for all cell tracks. The user can replace this data file with own data files.

After reading the data, a window pops-up, which shows the data plotted in 3D. The data are then transformed via unwrapping. In the same pop-up window the procedure of the algorithm can be followed, i.e. the data are shifted, grouped, unwrapped in the first dimension (rainbow colored, still curved surface), followed by unwrapping in the second dimension resulting in transformed data points that lie in on a flat 2D surface (grey plotted points).

After the data transformation took place, the R script analyses the initial data transformed via xy-projection as well as the data transformed via unwrapping. For both data sets the bias and persistence angles are computed and plotted as histograms, overlaid with the estimated density of the resulting distributions (red lines).

If you run the *in vivo* example in the folder '*exampleCode_Unwrapping_3_inVivo'* an additional pop-up window will open, where the trajectories are plotted in the xy-projection and after unwrapping for comparison. In this example the analysis output of bias and persistence distribution differs slightly. Here, we plot additionally to the bias and persistence distributions, the transformed bias distributions. As explained further below, the bias angles can take values between –pi and pi. In principle, this distribution should be plotted on a circle, because angles generate circular distributions. We refrain from doing so, since the circular representation is harder to read. However, one should keep in mind that –pi is equivalent to +pi. We aim to highlight this by shifting our obtained bias distribution by –pi. In this way it becomes clear that the bias distribution obtained from the unwrapped data indicates two maxima (one at 0 and one at –pi), which shows that cells are biased towards the wound (0), but also in the opposite direction, i.e. away from the wound (-pi).

Finally, we provide for comparison the Jupyter notebook for the presented manifold learning technique on the example of the persistent random walk based on the same data set as the R routine for unwrapping *exampleCode_Unwrapping_2_PRW* (see previous section).

1.3. Tutorial: How to do manifold learning with Jupyter

Installation and Prerequisites.

The method is implemented in Python and makes use of the following packages: *NumPy*, *Pandas*, *scikit-learn*, *Matplotlib* and *Seaborn*

The Jupyter notebook can be installed following the instructions in the unwrapping examples above, except there is no need for the *R* kernels here. Note that the code can be executed in both Python 2.7 and 3.x.

The Jupyter notebook document is titled *Manifold_learning.ipnb* (part of Data S1) and can be found in the folder *Jupyter (Data S1)*. A detailed step-by-step installation guide is included within. For reference, an HTML version of the document *Manifold_learning.html,* which can be accessed using any web browser, is also provided in the same folder.

1.4. Timing

All provided methods are able to handle large data sets. The provided examples run in a couple of seconds. The Unwrapping was tested on a dataset with 3000 data points. This takes depending on the computer used (here: Mac OS 10.8, 2.7 Ghz Intel Core i7, 16 GB Memory) several seconds. The manifold learning methods are slightly slower, again depending on data set size and computer used. The provided example in the Jupyter notebook analyses a dataset with a broadly realistic size of 3000 data points. The example can be run under a minute on a standard workstation. The manifold learning computation is dominated by the calculation of the similarity matrix, which in turn scales as $O(N^2)$ where N is the number of data points. Therefore a dataset with 20-25K data points could be analyzed within approximately one hour. In practice, the limitations are defined by the computer equipment (e.g. processor, memory, disk space).

1.5. Methods in Brief

1.5.1. Random walks in Biology

There are different types of random walks that are commonly described in Biology. We can classify them into random walks that describe the step length distribution and random walks that describe the angular distributions. The definition of random walks via step length distribution is somewhat more frequently used. However, to investigate if a cell or a molecule is targeted in its movement, it is easier to look at angular distributions.

The most prominent random walk is **Brownian motion**. The angular distribution is isotropic, meaning that at each step a cell or molecule has equal probability to move in any direction. If we measure the angles between a motion vector (cell step) and a reference direction, we will find that the resulting angular distribution is flat (uniformly distributed). If, on the contrary, a cell has a specific target direction, then the cell has higher probability to move towards that target direction compared to all remaining directions. In this case we speak about a **biased random walk**. The expected angular distribution will have a peak at the angle which points towards the target direction. The remaining characteristics of the angular distribution of such biased random walk depend on the details of the exhibited walk, which are usually unknown. However, a commonly used description of the angular distribution is a wrapped normal distribution (a normal distribution wrapped around a circle to describe circular variables such as angles). The mean of the wrapped normal distribution indicates the bias direction and the variance indicates the strength of the bias. The lower the variance, the narrower is the distribution and the stronger is the exhibited bias. A further type of random walk frequently used to describe animal movement and cell migration is a **persistent random walk**. A cell exhibiting this type of walk has higher probability of moving in the same direction as in the previous step compared to changing its

direction. If we measure the angles between consecutive motion vectors (consecutive cell steps) we will observe a peak at *0*, i.e. no change of direction. As for the biased random walk, the persistent random walk can also be described using a wrapped normal distribution with 0 mean and a variance which indicates the strength of the persistence (the lower the variance the stronger the persistence).

All three types of walks have been described for migration of immune and other cells, migration of animals, and movement of molecules inside the cell. Often a mix of these three types is observed.

1.5.2. Analyzing cell migration data

Cell migration trajectories are often extracted from confocal time-lapse microscopy imaging data. Recent advances allow researchers to collect such data even *in vivo* in living animals. Examples include imaging of macrophage, neutrophils and cancer cell migration in zebrafish tail fin, flanks, gills or yolk; imaging of stem cells and hematopoietic cells in mouse bone marrow; imaging of haemocytes in various stages and organs of drosophila; imaging of migrating neutrophils on the surface of the heart and many more.

While these data contain potentially a huge amount of new information about the underlying biological processes *in vivo*, their correct analysis buries a vast range of challenges and one of them we highlighted in this study: the movement of cells on curved surfaces.

In order to extract information about bias and persistence from observed cell trajectories, we have to compute two types of angles: (i) the angle α between a fixed reference direction and the cell motion vector and (ii) the angle β between two consecutive motion vectors. While the first angle α helps us to detect potential bias direction, the later angle β helps us to measure the strength of persistence (as described in the previous section). If the movement of the cell is restricted to a curved surface, then directly measuring the angles α and β based on the original (untransformed) data will provide us with artifacts, which in some cases could mimic a target bias where in reality there is none. In order to still be able to extract bias and persistence information from such data, we need to either transform the trajectory data in such way that they lie on a flat surface (and then apply the standard analysis tools), or use some methods to learn the exact surface (manifold) and compute the angles on such manifold. Either way, the aim is to remove any artifacts that appear through curved surfaces from the analysis. The first solution can be obtained via unwrapping; the second brings us to the field of manifold learning.

1.5.3. Unwrapping trajectory data

As mentioned in the previous section, unwrapping trajectory data aims to transform data from a curved surface so that they lie on a flat surface.

More specifically, the Unwrapping method is fitting several ellipses to the observed data points. These ellipses can then be unrolled onto a 2D surface. The basic idea behind this method is rather simple and intuitive: Imagine our cells are migrating on the peel of an orange, which is clearly a curved surface describing a sphere or an ellipsoid. The aim is now to peel the orange in such way that we can lay the peel on the flat table and still conserve the characteristics of the cell trajectories. We are here interested to conserve directional characteristics, more than distances. The resulting transformed cell trajectories can now be analyzed with the commonly used tools.

Unwrapping is best suited for 3D objects that have a rather small intrinsic and convex curvature. This means before this method is applied, we already have an idea of the true geometry.

1.5.4. Manifold learning

Manifold learning refers to a diverse suite of methods that aim to generalize well-known *linear* dimensionality reduction methods – Principal Component Analysis (PCA), Independent Component Analysis (ICA), Linear Discriminant Analysis (LDA) – to account for non-linear features in the data.

The key assumption underlying dimensional reduction methods – linear and non-linear – is that the 'true' number of degrees of freedom is lower than the apparent dimensionality of the data. The problem addressed in this paper gives the simplest and perhaps the most explicit illustration: we have point-cloud data in three dimensions constrained to lie on two-dimensional surfaces, which may or may not be flat.

The study of smooth curved spaces belongs to the mathematical field of *differential geometry*. There is an intuitive idea underlying this field: at small enough scales, every local patch of a surface can be approximated by a flat surface; a curved manifold is then simply an overlapping patchwork of (small) flat spaces. This is the approach adopted by most manifold learning algorithms – that is, to identify local neighborhoods of data points, treat these as linear spaces, and then find some 'optimal' method of joining these together into a flat global representation. A necessary requirement for these methods to work well, therefore, is that the density of the data points is high enough to allow one to consider small linear patches.

If one starts to consider distances between points and angles between vectors, then we augment this description of the manifold with a *metric,* which is a mathematical object that, loosely speaking, provides a local specification of lengths and angles. A manifold with a metric is known as a *Riemannian manifold* and is the object of study in *Riemannian geometry*.

Manifold learning algorithms do not explicitly preserve the metric information. However one can augment these methods by extracting the metric at each of the data points. This turns outs to be essential for obtaining accurate directional statistics, as we show in this paper.

1.6. Image processing and cell tracking.

Imaging resulted in image stacks with dark background and fluorescent cells. The image processing was done in R using the package EBImage [1]. The information of the cells was extracted automatically from the images using an edge detection method. A manually set threshold of the light intensity was used per image stack. Each detected cell was described as an object with the coordinates of its geometrical center indicating the cell location and the time the cell was observed. The cells were tracked and reconstructed over the z-stack using a surface algorithm. The surface algorithm was then applied to track reconstructed cells over time, which is based on the shortest distance between cells from two consecutive images. We excluded all cell trajectories that included time points in which the cell was located at the edge of the image. Extracted cell tracks were reoriented, so that the center of the imaged object (embryo or yolk syncytium) was positioned in the center of the coordinate system ($x = y = z = 0$) for further processing.

1.7. Dimensional reduction: from linear methods to Riemannian manifold learning.

In this section we provide the theoretical background to the methods employed in the paper, including the unwrapping method outlined in Section 1. The challenge of describing and visualizing the geometry of embedded curved surfaces is commonly encountered in physics [2], computer vision, and machine learning [3] tasks. The techniques used are those from differential geometry or, more specifically, Riemannian geometry. For the sake of completeness and consistency, we adopt this more formal mathematical description. We provide a brief introduction to the essential topics; for more details, we refer the reader to [2]. Let $\mathcal{M}$ be a smooth m-dimensional manifold and g the Riemannian metric defined for every point $p \in \mathcal{M}$. For a smooth manifold $\mathcal{N}$ with $\dim(\mathcal{N}) \equiv n \geq m$, let $f: \mathcal{M} \rightarrow \mathcal{N}$ be an isometric embedding, *i.e.* for all $p \in \mathcal{M}$ and tangent vectors $u, v \in T_p\mathcal{M}$

$$\langle u, v\rangle_{g_p} = \langle df_p(u), df_p(v)\rangle_{h_{f(p)}},$$

here $\langle\, ,\rangle_{g_p}$ is the inner product on the tangent space $T_p\mathcal{M}$, $g_p \equiv g(p)$, and h the metric defined for every point $q \in \mathcal{N}$. $df_p: T_p\mathcal{M} \to T_{f(p)}\mathcal{N}$ is the Jacobian of f at p.

For a dataset $D = \{q_1, \dots , q_N\}$ of points in $\mathcal{N}$, dimensional reduction is the task of inferring the inverse map $f^{-1}: \mathcal{N} \to \mathcal{M}$. For many purposes it is often sufficient to infer the corresponding images $\{x(p_1), \dots, x(p_N)\}$ for $q_i = f(p_i)$ and in some coordinate chart $x: \mathcal{M} \to \mathbb{R}^m$. Throughout this paper, we restrict ourselves to $\mathcal{N} \subset \mathbb{R}^3$ (*i.e.* 3D imaging data). If $\mathcal{M} \subset \mathbb{R}^{1,2}$ then we can use linear dimensional reduction methods. Here we consider two linear and three non-linear methods.

Projection into the XY –plane. A trivial and linear dimensional reduction method is the simple projection onto some pre-defined set of coordinate axes. We assume, without loss of generality, that these are the first m coordinates; in two dimensions, these are the *X*- and *Y*-axes, hence the name. Then for $i = 1, \dots, N$, we simply have

$$[x(p_i)]_a = [q_i]_a, \quad a = 1, \dots, m,$$

where the a subscript is the coordinate index.

Principal component analysis. Instead of pre-specifying the axes, we can perform linear dimensional reduction via principal component analysis (PCA). Let $C = \frac{1}{N-1}\sum_{i=1}^{N}(q_i - \bar{q})(q_i - \bar{q})^T$ be the sample covariance matrix, with the mean $\bar{q} = \frac{1}{N}\sum_{i=1}^{N} q_i$. Then for the rotation matrix $R = (e_1|e_2|e_3) \in O(N), e_1, e_2, e_3$ the eigenvectors of C in decreasing order of their respective eigenvalues, we have

$$[x(p_i)]_a = [Rq_i]_a, \quad a = 1, \dots, m.$$

Unwrapping. We introduce a method to map data points on a 2D convex surface onto a subspace of $\mathbb{E}^2$, the 2D Euclidean space, which we call the Unwrapping method. This method is a particularly effective approximation for surfaces of small intrinsic curvature (e.g. a thin cigar-shaped surface, a small patch on a large curved surface, etc). The unwrapping happens in two steps, both of which involves fitting a series of 1D ellipses to the data. The first step is a transformation, which removes the intrinsic curvature of the surface while seeking to maintain the geometrical relationships between the points (i.e. distances, angles, etc). The second step simply unwraps the transformed surface onto a flat 2D surface.

Let (x_i, y_i, z_i) represent the 3D coordinates of the data point q_i. If we approximate the dataset as points on a subspace of an ellipsoid, we choose to align our coordinate system such that the largest radius of the ellipsoid is described by the *x*-axis and the second largest radius is the *y*-axis. Next the data points are clustered into n equal-sized bins along the x-axis. For each cluster *c* we fit an ellipse E_c as the locus of the equation

$$\frac{y^2}{r_y^2} + \frac{(z-m_z)^2}{r_z^2} = 1,$$

for radii r_y, r_z and the *z*-coordinate m_z of the midpoint $(\overline{x_c}, 0, m_z)$, with $\overline{x_c}$ the mean *x*-coordinate of the cluster *c*. The ellipse is then unrolled onto a straight line parallel to the *y*-axis with $x = \overline{x_c}$ and $z = z_c^{max}$ the maximum *z*-coordinate value of the points in cluster *c*. This then guides the first transformation of the points $(x_i, y_i, z_i) \to (x_i', y_i', z_i')$ as follows. We keep the *x*-coordinate fixed, *i.e.* $x_i' = x_i$. As for the *z*-coordinate, because, in general, the data points do not lie on the ellipse (*i.e.* $\notin E_c$), we let z_i' be equal to the difference in distances to the center of E_c from q_i and the point on E_c along the line joining q_i and the centre. It can be shown that

$z_i' = |q_i - q_i^E| \equiv d_i,$

where q_i^E is a point with components

$$[q_i^E]_1 = x_i$$
$$[q_i^E]_2 = \frac{y_i r_z^2}{2(z_i - m_z) r_y^2} - \sqrt{\frac{y_i^2 r_z^4}{4(z_i - m_z)^2 r_y^4} + \frac{y_i r_z^2}{z_i - m_z}}$$
$$[q_i^E]_3 = \sqrt{\frac{r_y^2 r_z^4 - r_z^2([q_i^E]_2)^2}{r_y^2}}$$

To determine y_i', we define a point-specific ellipse E_i with the same centre $(\overline{x_c}, 0, m_z)$ as E_c but with radii $r_{z,i} = r_z + d_i$ and $r_{y,i} = r_{z,i}\frac{r_y}{r_z}$. Then if $q_{i,z}$ is the intersection of the E_i with the *xy*-plane, y_i' is then the arc length of E_i between $q_{i,z}$ and q_i. Repeating this transformation for all *n* clusters results in the first unwrapping of the data points.

For the second unwrapping the same procedure is repeated on the transformed data set giving $q_i' \to q_i''$, but with the variable swap $x \leftrightarrow y$. Note, if all data points $\{q_i\}_{i=1}^N$ lie strictly on an ellipsoidal surface then $z_i'' = 0$ for all $i = 1, \ldots, N$. An example tutorial is provided in suppl. material.

Manifold learning. *Manifold learning* refers to a class of non-linear dimensional reduction methods that seek to recover the geom etry of the low-dimensional manifold. These include ISOMAP [4], Locally Linear Embedding (LLE) [5], and Laplacian Eigenmaps [6], amongst several others. In every case, the metric on M is a global Euclidean metric, *i.e.* $g_{ab} = \delta_{ab}$, where δ the kronecker delta or identity matrix and *a, b* the coordinate indices. We refer to these approaches as *Euclidean manifold learning*.

In this paper we have employed LLE in our simulation and analysis of real data. LLE is based on the expectation that given a sufficiently large data set, each data point and its closest neighbors lie on a locally linear patch of the surface. The algorithm has two steps: 1. Expressing each higher dimensional data point as a linear combination of its neighbors, and 2. Obtaining a set of lower dimensional coordinates given relations above. In both steps, we proceed by minimizing the mean square errors of the data points from their linear reconstructions.

Our motivation for adopting LLE comes from both its intuitive approach to dimensionality reduction (locally linear patches) and also its effectiveness in providing an isometric reduction for surfaces with no intrinsic curvature, e.g. the surface of a cylinder or data points on a cylindrical 'swiss-roll'. Furthermore it is also widely used in the machine learning community. In this paper we have used the implementation of LLE in the Scikit-learn Python machine learning package.

Riemannian manifold learning. Riemannian manifold learning aims to augment the set of coordinates $\{x(p_i)\}_{i=1}^N$ with the corresponding set of local metric values, *i.e.*

$$\{(x(p_i), \delta_{ab})\}_{i=1}^N \to \{(x(p_i), [g(p_i)]_{ab})\}_{i=1}^N,$$

where $a, b = 1, \ldots, m$ label the metric components. By definition, g is symmetric and positive semidefinite. In this setup, one can recover the precise geometrical information of the embedded manifold. In this paper, we have adapted the LEARNMETRIC5 [3] algorithm to recover the 2D coordinates and the corresponding metric components from 3D data points.

For certain applications, such as computing the geodesics (see below), there is a need to derive metric values for out-of-sample points on $\mathcal{M}$. The metric $g(x)$ for $x \notin \{x(p_i)\}_{i=1}^N$ are approximated in two steps. First we perform a regression for each of the $m(m+1)/2$ unique components of g. In this paper we used the

implementation of Gaussian Processes in the Scikitlearn *Python* machine learning package. Second we satisfy the positive semidefinite constraint by replacing the matrix $g(x)$ with the nearest positive semidefinite matrix as measured by the Frobenius Norm. We implement this using the approximation method of Higham (2002) [7].

1.8. Extraction of geometrical information

All the relevant geometrical information of interest can be extracted from the metric. The angle between the two vectors u, v is given by

$$\theta = cos^{-1}\frac{\langle u,v\rangle_{gp}}{\langle u,u\rangle_{gp}\langle v,v\rangle_{gp}}.$$

The geodesic $\gamma: \mathbb{R} \to \mathcal{M}$, is the path of extremal length and is the solution to the set of Hamiltonian equations. With slight abuse of notation writing $x(\gamma(t)) \equiv x(t)$, these equations are

$$\dot{x}^a = \frac{\partial H}{\partial r_a} = g^{ab} r_b,$$
$$\dot{r}^a = -\frac{1}{2}\frac{\partial g^{bc}}{\partial x_a} r_b r_c,$$

where r_a is the conjugate momenta to x^a, g^{ab} the components of the inverse metric, $\dot{x} \equiv dx/dt$, and the Hamiltonian

$$H = \frac{1}{2} g^{ab} r_b$$

The bias direction from cell at a given time to a point source of attraction is given by the tangent vector u' to the geodesic connecting the two points. Therefore we solve the geodesic equations (9) for $x(t)$ under the constraints

$$x(t = 0) = x(p_1), \;\; x(t = 1) = x(p_2).$$

One approach is to simulate these geodesics from p_1 via, say, the simple Euler method [8] and find the initial vector that generates the points on the geodesic that intersects p_2. However this seemingly straightforward process is highly sensitive to errors in the out-of-sample metric approximations – the errors compound and one often ends up with unstable trajectories. In this paper, we implement a more stable and efficient discrete approximation as follows.

We first overlay a grid over the learned manifold. Here we fix the grid dimensions to 200 x 200. Next, using the Gaussian Process regression method described above, we obtain the metric values at every grid vertex and consequently the lengths of the sides of the cells across the grid. Then we approximate our geodesics between the point of interest and the bias point by the grid path that minimizes the total length. We usd Dijkstra's algorithm to accomplish this step. Finally, we approximate the initial vector by performing a simple linear regression on the first few vertices in our discretized geodesic path.

1.9. Directional Statistics.

The straightness index, D, is commonly used to investigate cell migration strategies and it is defined as $D = \frac{|x_0, x_T|}{l}$, where x_0 is the position of the cell at time 0, x_T is the position of the cell at time T, $|x_0, x_T|$ indicates the shortest distance between x_0 and x_T and l is the actual length of the path the cell took from x_0 to x_T . For most applications the shortest path between start and end point is simply the Euclidean distance, however, for curved surfaces the metric needs to be learned.

Another way of describing cell migration tracks is by determining their bias towards a specific target (source of

attractant, like wounds or other cell types) and their persistence. We define the persistence of a cell as the probability of the cell moving at time t in the same direction as at time $t-1$. Therefore, we need to compute the angles (β_t) between two motion vectors, which will result in a characteristic distribution. The wrapped normal distribution has been successfully used to describe persistent movement of cells. The probability density function is defined as

$$N_w(\beta_t|\mu,\sigma) = \frac{1}{\sigma\sqrt{2\pi}}\sum_{k=-\infty}^{\infty} \exp\left(-\frac{(\beta_t-\mu+2\pi k)^2}{2\sigma^2}\right),$$

where μ is the mean and σ is the standard deviation. In the case of persistence we have $\mu = \beta_{t-1}$. We can then define the strength of the persistence p as $\sigma = -2\log(p)$. A cell that is highly persistent has a p close to 1, while a cell that is not persistent at all has a p of 0, in which case the wrapped normal distribution becomes a wrapped uniform distribution. The bias of a cell is also described by an angular distribution. Here, we compute the angle (α) between a motion vector of a cell and the vector that points from the cell towards the target. Again, we apply the wrapped normal distribution with the bias parameter b (instead of p) describing the strength of the bias and $\mu = 0$.

1.10. Simulation of random walks on ellipsoids

The ellipsoid is defined as the set of points satisfying

$$\frac{x^2}{a^2} + \frac{y^2}{b^2} + \frac{z^2}{c^2} = 1,$$

with x, y, z the 3D cartesian coordinates, and a, b, c the three shape parameters. We consider several different ellipsoid shapes with a/c ratios from the set {1, 0.66, 0.5, 0.4, 0.33, 0.1} where $a = b$ throughout. We use the 2D parameterization

$$x = a\cos\mu\sin\nu,$$
$$y = b\sin\mu\sin\nu,$$
$$z = c\cos\nu,$$

with metric components

$$g_{\mu\mu} = (\sin^2\nu)(a^2\sin^2\mu + b^2\cos^2\mu),$$
$$g_{\mu\nu} = g_{\nu\mu} = \sin\mu\cos\mu\sin\nu\cos\nu,$$
$$g_{\nu\nu} = (\cos^2\nu)(a^2\cos^2\mu + b^2\sin^2\mu) + c^2\sin^2\nu.$$

We simulate 400 random walks on each of the ellipsoids as follows: starting from the initial point $(\mu_0, \nu_0) = \left(\pi, \frac{\pi}{2}\right)$, we randomly select an initial angle of motion $\theta_0 \sim B(\theta, \theta_0')$, where B is the bias angle distribution with θ_0' the direction to the bias source at time-step index t; θ_0' is determined following the minimization procedure described above and, in turn, defines an initial tangent vector. The particle moves along the geodesic generated by this vector with random step length taken from a χ^2-distribution with mean$k = 2$; the absolute lengths are scaled with a constant factor in the range 0.015 – 0.25 to ensure that the trajectories cover the ellipsoid. At each subsequent time step $t > 1$, the particle changes direction and takes an angle according to a weighted distribution

$$\theta_t = wB(\theta, \theta_{t-1}') + (1-w)P(\theta - \theta_t^{old}),$$

where P is the persistence angle distribution and $\theta_t^{old}$is angle of motion prior to changing directions at time step t. Both P and B are either uniform distributions in the range $(0,2\pi]$ or wrapped normal distributions with parameter $\sigma = 1.2$, depending on the type of random walk being simulated. For the bias persistent random walk, we fix the weight parameter to be $w = 0.5$. We repeat each path for 20 time steps, giving 21 trajectory points per path.

2. Supplemental figure, movie and data legends

Figure S1. Related to Figure 2. Performance comparison of xy-projection, the unwrapping method and manifold learning methods. Shown are the exact distributions (black) that describe bias and persistence for 3 types of random walks: (A) Brownian motion, (B) biased random walk and (C) persistent random walk. Cell trajectories were simulated on the surface of ellipsoids with different ratios of its radii (a/c ratio = 0.1, 0.25, 0.33, 0.5, 0.66 and 1.0). For each scenario we compute the distributions based on the xy-projection, the unwrapping method and the two manifold learning methods. The two manifold learning methods are not able to deal with the two lowest a/c ratios and where left out. The reason is that these ellipsoids were too elongated, so that the manifold learning methods treated the data as if they were located in a slim plane. (E) Shown are the exact persistence angle distribution (black), the persistence angle distribution computed from the 3D vectors (green) and the persistence angle distribution resulting from unwrapping the data into a non-curved surface (red). The underlying data are persistent random walk trajectories simulated on an ellipsoid with radii ratio a/c = 0.33. (F-G) Application of the unwrapping method (orange) to neutrophil cell tracks extracted from the epidermis overlying the yolk syncytium of a zebrafish and its comparison to the xy-projection (blue) and principle component analysis (green). The epidermis was wounded with a laser before image acquisition. (Corresponds to Figure 2F)

Figure S2. Related to Figure 2. Performance measurements of the different methods. We computed the Kolmogorov-Smirnov distance (ks-distance) between the true angular distributions and the extracted angular distributions using xy-projection, unwrapping, Euclidian manifold learning and metric manifold learning. We considered the same data generated for the random walk models described in Supplemental Figure 1A-C. The smaller the ks-distance is, the better is the performance of the methods.

Figure S3. Related to Figure 2. Shown are the 2D projections of cell tracks extracted from Drosophila embryos (colored tracks) after applying (A) *xy*-projection, (B) unwrapping and (C) manifold learning. The green, orange, dark blue and red tracks are highlighted for easy comparison between the methods. The grey lines in (B) and (C) are for comparison with the *xy*-projection as they are the same tracks using the *xy*-projection.

Figure S4. Related to Figure 2. Manifold learning with more complex data. (A) We simulated random walk cell trajectory data on a complex surface. Shown is the 3D representation of these data (blue) with the xy-, xz- and yz-projections (grey). This example is too complex to be successfully transformed via unwrapping, but can be dealt with manifold learning techniques. The trajectories display a Brownian motion type random walk, where the bias and persistence distributions are expected to be flat. (B) We analyze the data in the xy-projection and compare this to the Euclidian manifold learning algorithm and the metric manifold learning algorithm. As can be seen in supplemental figure 4B, the xy-projection induces extreme artifacts. The bias distribution is well extracted from both manifold learning methods, while the persistence distribution is only correctly extracted using the metric manifold learning algorithm, highlighting the need to accurately learn the metric of the data

Movie S1. Related to Figures 1 and 2. Shown are example raw data for the unwounded *Drosophila* embryo data set analyzed in figures 1A, 2E and Supplemental Figure 1. Time-lapse movie of the dynamic behavior of *Drosophila* immune cells (heamocytes) in unwounded tissue. Epithelial cells are labeled using E-cadherin-GFP (green cell outlines), immune cell nuclei are labeled using nuclear Red-Stinger (red) and immune cell cytoplasm using cytoplasmic GFP (green) both driven by *srp-Gal4*.

Movie S2. Related to Figures 1 and 2. Shown are example raw data for the wounded zebrafish data set analyzed in figures 1B and 2F. Time-lapse movie of the dynamic behavior of zebrafish immune cells (neutrophils) in laser-induced wounded tissue. Immune cells are labeled using cytoplasmic dsRed (red) driven by the lysozyme C (*lyz*) promoter.

Movie S3. Related to Figures 1 and 2. Shown are the haemocyte cell tracks extracted from *Drosophila* embryos in 3D. The different rotation angles show the curvature of the space the haemocytes are migrating in.

Movie S4. Related to Figures 1 and 2. Shown are the neutrophil cell tracks extracted from zebrafish embryo.

The epidermis overlying the yolk syncytium was wounded. The location of the wound is indicated by a light blue dot.

Data S1. Related to Experimental Procedures. This folder contains the two Jupyter notebooks for Unwrapping and Manifold learning. Furthermore it includes the two websites for both methods. Example data are stored in the folder 'SimulationData'.

Data S2. Related to Experimental Procedures. This folder contains all described R scripts and the provided example data in order to perform Unwrapping on simulated and *in vivo* data.

3. Supplemental figures.

Figure S1. Related to Figure 2.

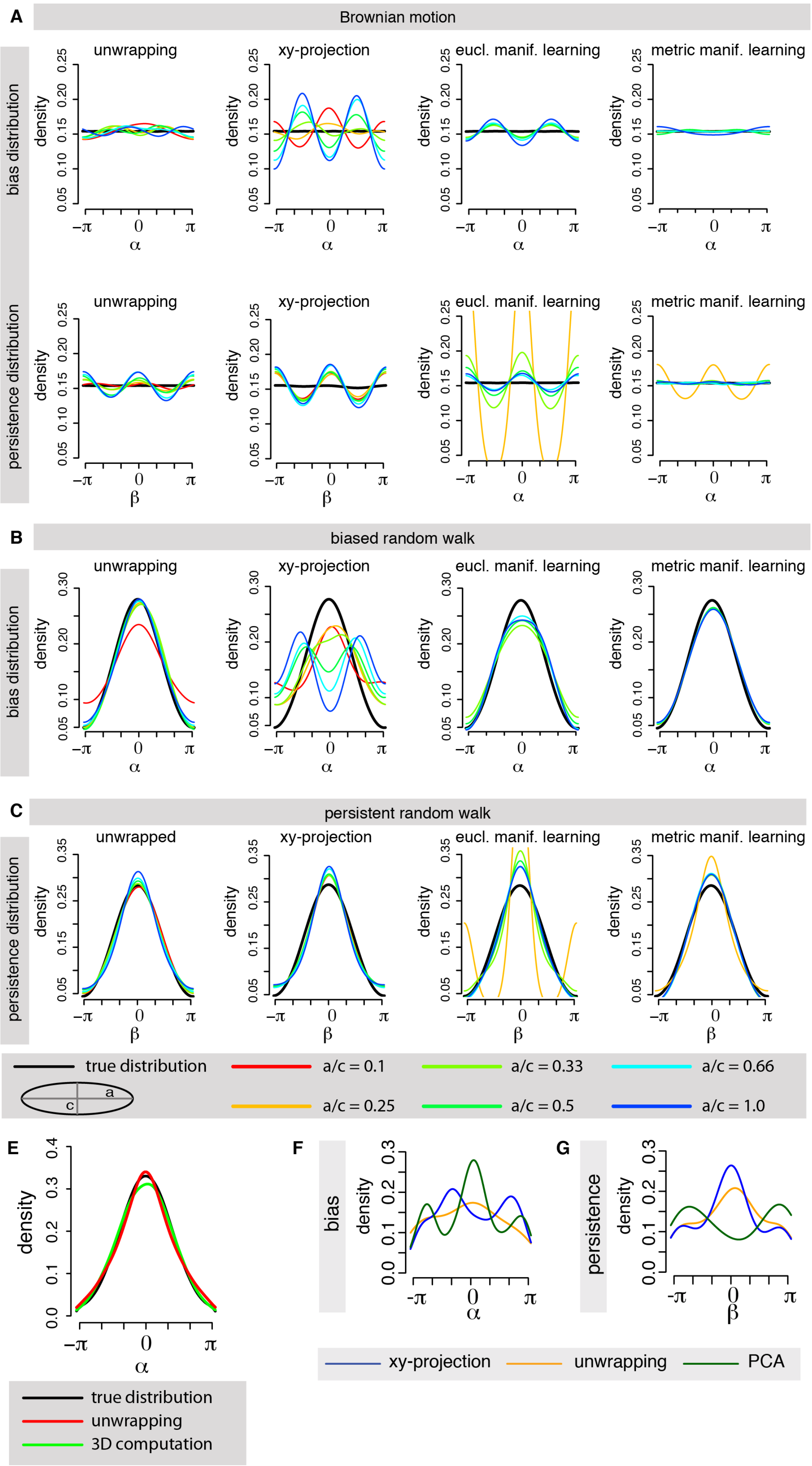

Figure S2. Related to Figure 2.

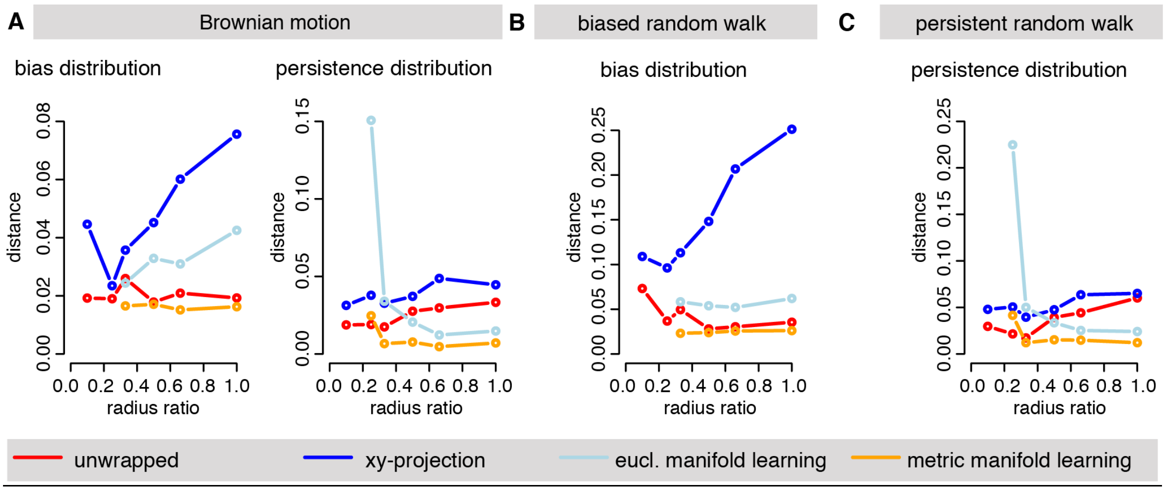

Figure S3. Related to Figure 2.

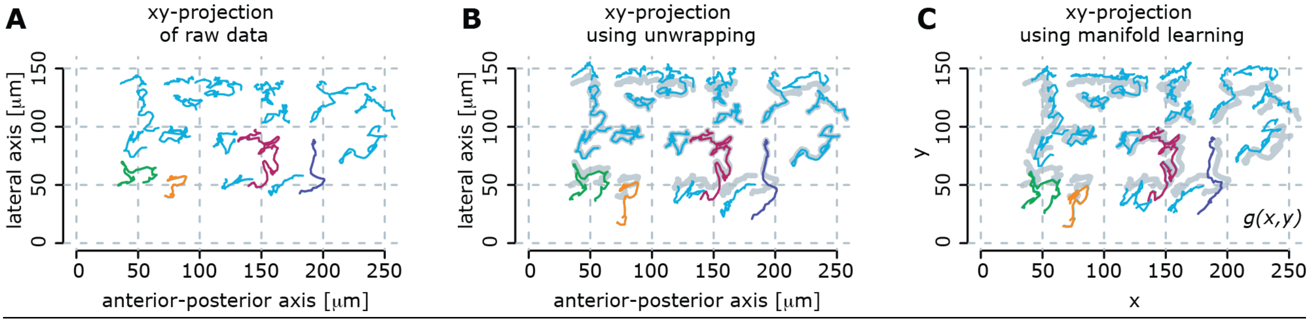

Figure S4. Related to Figure 2.

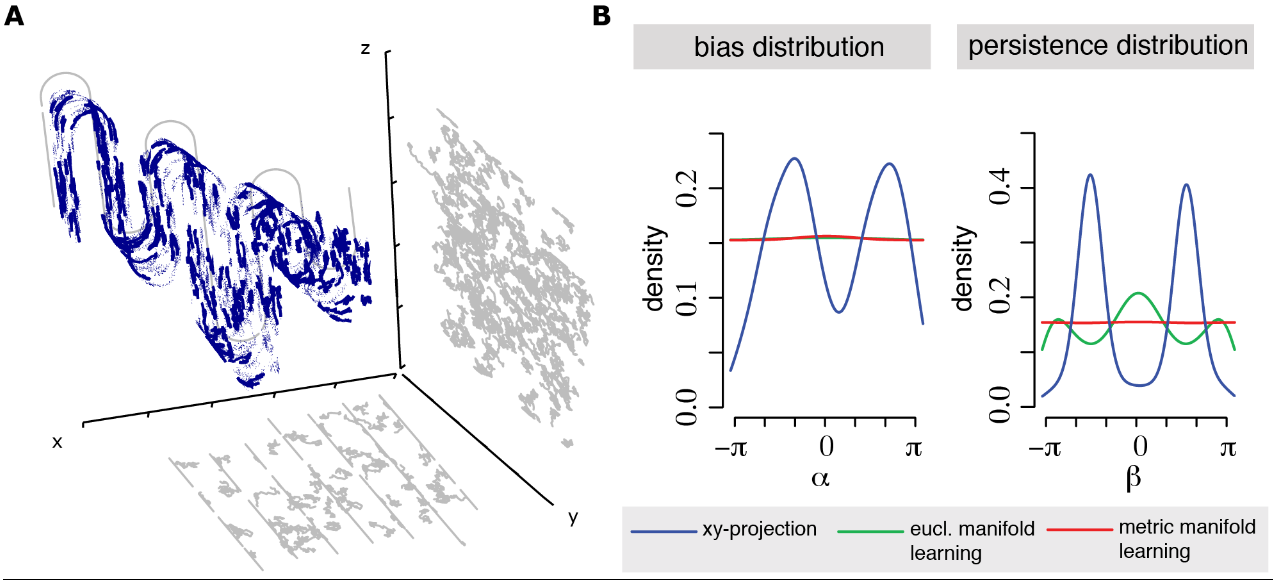

4. Supplemental References.

1. Pau, G., Fuchs, F., Sklyar, O., Boutros, M. & Huber, W. Ebimage - an r package for image processing with applications to cellular phenotypes. Bioinformatics 26, 979–81 (2010).
2. Nakahara, M. Geometry, Topology and Physics, Second Edition (CRC Press, 2003).
3. Perraul-Joncas, D. & Meila, M. Non-linear dimensionality reduction: Riemannian metric estimation and the problem of geometric discovery. arXiv.org (2013). 1305.7255v1.
4. Tenenbaum, J. B., de Silva, V. & Langford, J. C. A global geometric framework for nonlinear dimensionality reduction. Science (New York, N.Y.) 290, 2319–+ (2000).
5. Roweis, S. T. & Saul, L. K. Nonlinear dimensionality reduction by locally linear embedding. Science (New York, N.Y.) 290, 2323–+ (2000).
6. Belkin, M. & Niyogi, P. Laplacian eigenmaps for dimensionality reduction and data representation. Neural Computation 15, 1373–1396 (2003).
7. Higham, N. J. Computing the nearest correlation matrix - a problem from finance. IMA Journal of Numerical Analysis 22, 329–343 (2002).
8. Leimkuhler, B. & Reich, S. Simulating Hamiltonian Dynamics (Cambridge University Press, 2004).
