# Supplementary figures and images for: Accurate Reconstruction of Cell and Particle Tracks from 3D Live Imaging Data
Source: Cell Syst. 2016 Jul 27;3(1):102–7. doi: 10.1016/j.cels.2016.06.002 (PMC4963212; doi:10.1016/j.cels.2016.06.002)

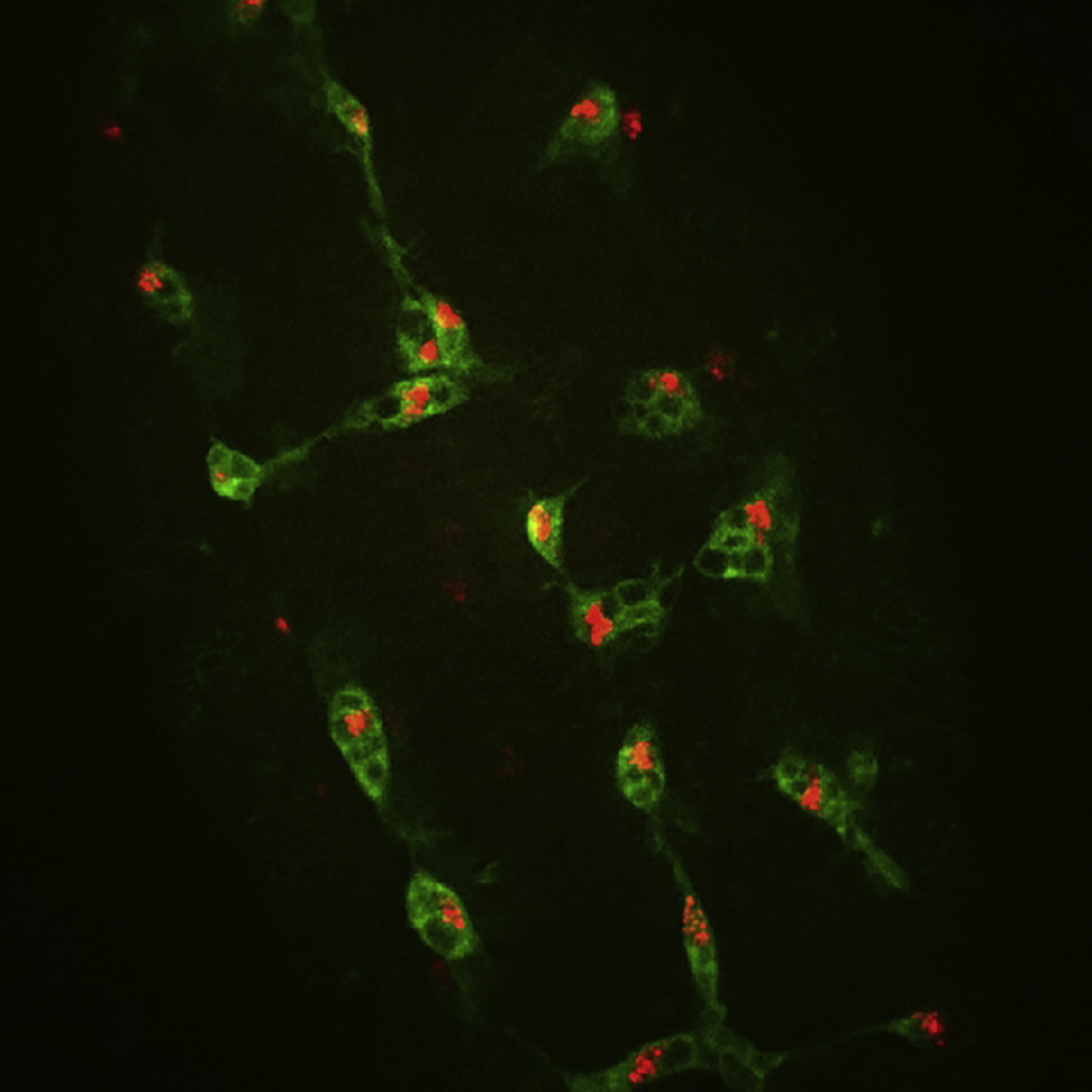

Supplement: Movie S1. Shown Are Example Raw Data for the Unwounded Drosophila Embryo Data Set Analyzed in Figures 1A, 2E, and S1, Related to Figures 1 and 2 — Time-lapse movie of the dynamic behavior of Drosophila immune cells (heamocytes) in unwounded tissue. Epithelial cells are labeled using E-cadherin-GFP (green cell outlines), immune cell nuclei are labeled using nuclear Red-Stinger (red) and immune cell cytoplasm using cytoplasmic GFP (green) both driven by srp-Gal4. [file mmc2.jpg]

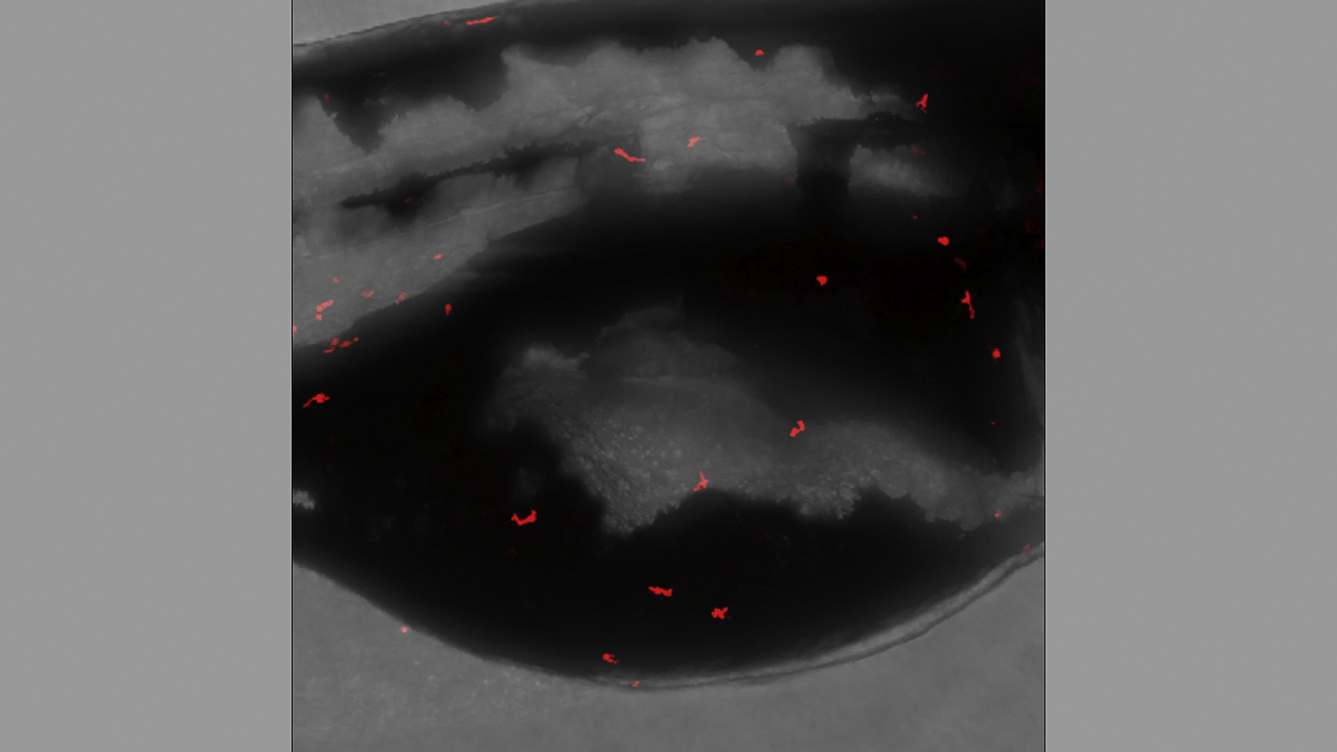

Supplement: Movie S2. Shown Are Example Raw Data for the Wounded Zebrafish Data Set Analyzed in Figures 1B and 2F, Related to Figures 1 and 2 — Time-lapse movie of the dynamic behavior of zebrafish immune cells (neutrophils) in laser-induced wounded tissue. Immune cells are labeled using cytoplasmic dsRed (red) driven by the lysozyme C (lyz) promoter. [file mmc3.jpg]

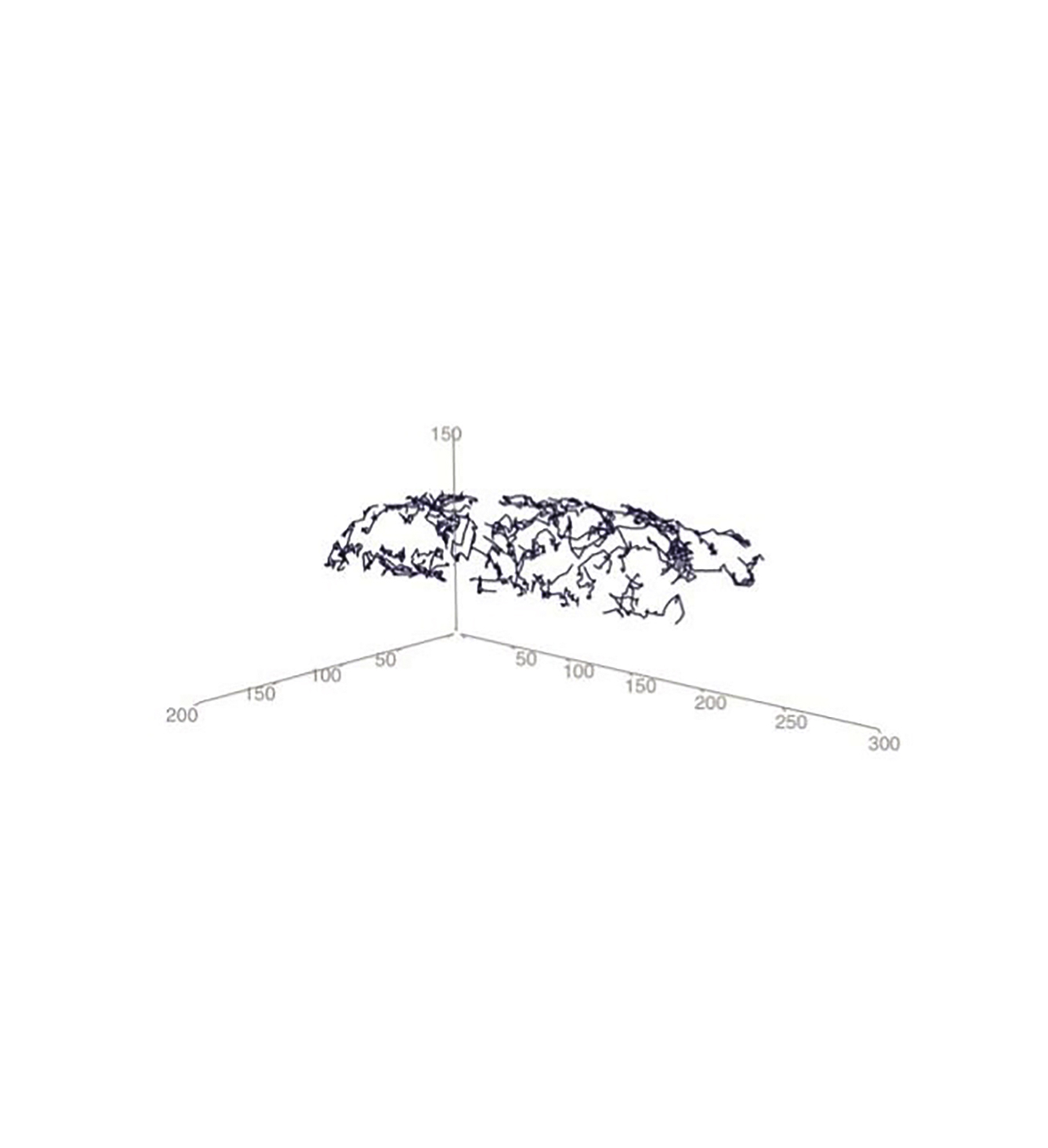

Supplement: Movie S3. Shown Are the Haemocyte Cell Tracks Extracted from Drosophila Embryos in 3D, Related to Figures 1 and 2 — The different rotation angles show the curvature of the space the haemocytes are migrating in. [file mmc4.jpg]

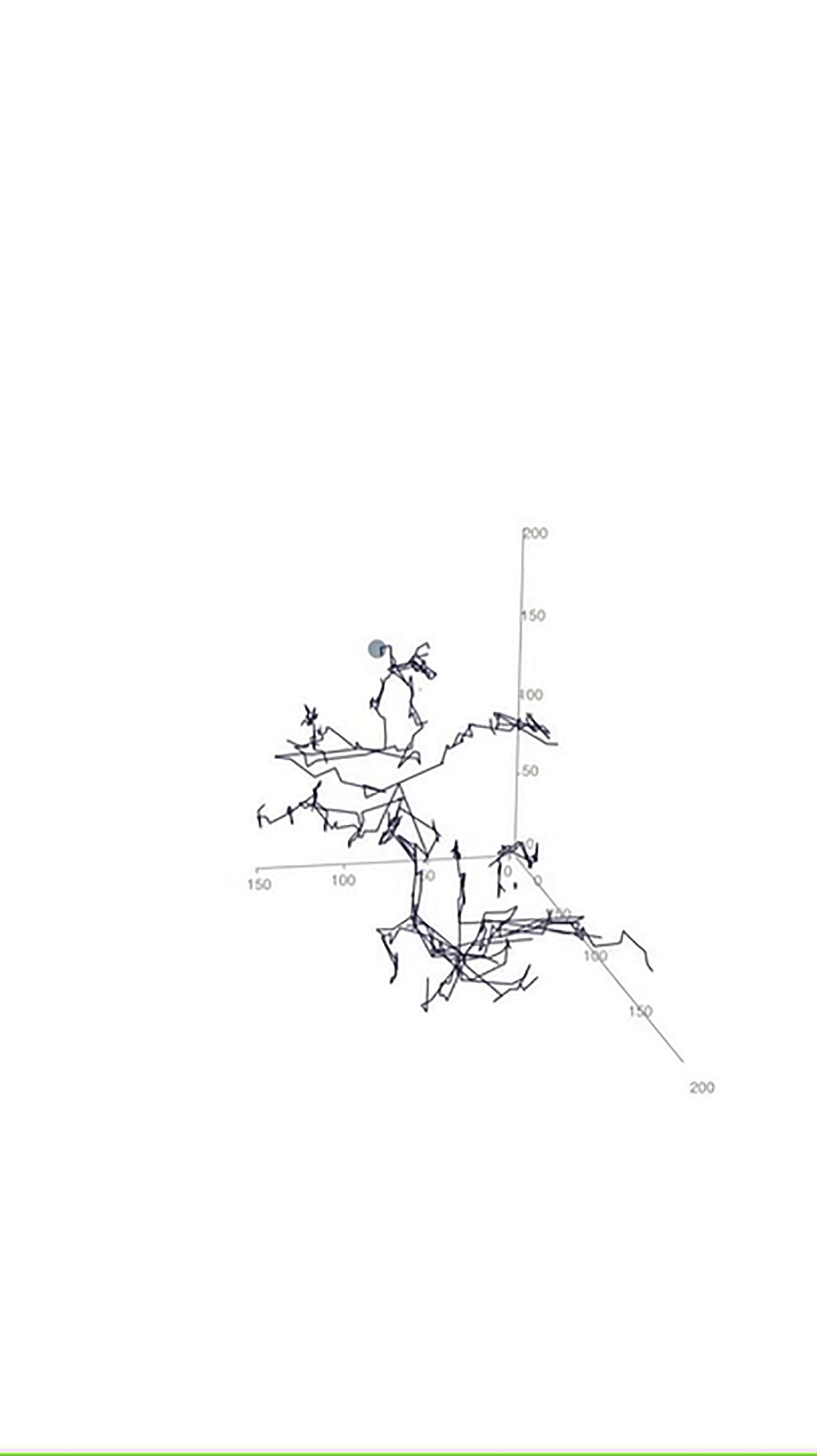

Supplement: Movie S4. Shown Are the Neutrophil Cell Tracks Extracted from Zebrafish Embryo, Related to Figures 1 and 2 — The epidermis overlying the yolk syncytium was wounded. The location of the wound is indicated by a light blue dot. [file mmc5.jpg]
