# Supplementary material for: Accurate Reconstruction of Cell and Particle Tracks from 3D Live Imaging Data
Source: Cell Syst. 2016 Jul 27;3(1):102–7. doi: 10.1016/j.cels.2016.06.002 (PMC4963212; doi:10.1016/j.cels.2016.06.002)
Supplement: Data S1. This Folder Contains the Two Jupyter Notebooks for Unwrapping and Manifold Learning, Related to Experimental Procedures — Furthermore it includes the two websites for both methods. Example data are stored in the folder “SimulationData.” [file mmc6.zip › Jupyter/Manifold_learning.html]

Manifold\_learning


# Unwrapping *in vivo* live imaging data¶

## 2D manifold learning in Python¶

This document provides a step-by-step description of the Riemannian manifold learning approach to unwrapping 3D imaging data. For illustration purposes, the method is applied to a simulated data set of random walks on the surface of an ellipsoid.

---

## Install the dependencies¶

The method is implemented in Python 2.7 and makes use of the following packages (with the specific uses in parentheses):

- NumPy (linear algebra)
- pandas (data file handling)
- scikit-learn (manifold learning)
- matplotlib and Seaborn (visualisation)

These can be installed either individually following the instructions in the links above, or all together in a free Python distribution such as Anaconda. Note that the code has been written to be compatible with both Python 2.7 and Python 3.x.

To run the Jupyter notebook you need:

- a web browser (Chrome, Safari, etc)
- an installation of the Jupyter notebook. The step-by-step installation instructions for Mac, Linux and Windows can be found here.

The simulated data is provided in the folder 'SimulationData' and this folder should be placed in the same parent folder as this notebook.

---

## Outline¶

The remainder of the document is organised as follows:

1. Import libraries
2. Define functions
3. Load simulated 3D random walk data
4. Compute global 2D chart coordinates
5. Perform metric learning
6. Find the empirical persistent angle distributions
7. Compare results against the generative (i.e. true) distribution

## 1. Import libraries¶

Import the external packages:

In [1]:

```
import matplotlib as mpl
import matplotlib.pyplot as plt
import numpy as np
import pandas as pd
import numpy as np
import seaborn as sns
import sklearn
import sklearn.manifold
```

## 2. Define functions¶

We first define the following three functions for learning the geometry:

- `constr_sim_matrix` : construct a similarity matrix
- `constr_laplacian` : construct the normalised graph Laplacian
- `metric_learn` : implement the metric learning algorithm (http://arxiv.org/pdf/1305.7255.pdf)

### 2.1 Similarity matrix¶

For a bandwidth $k\in \mathbb{R}^+$, the similarity matrix $K\_{ij}$ is defined via the truncated heat kernel
\begin{align}
K\_{ij} = & \exp\Bigl(-\frac{||p\_i-p\_j||^2}{k}\Bigr) &\quad (||p\_i-p\_j||^2 < k)\
&0 & (\text{otherwise}),
\end{align}
where $p\_i, p\_j\in\mathbb{R}^3$ are the 3D coordinate vectors representing data points $i$ and $j$ respectively.

In [2]:

```
def constr_sim_matrix(D, k):
    """Construct a truncated similarity matrix
    
    Parameters:
    D: (ndarray)
        The data array, with shape=(no. of samples, no. of coordinates)
    k: (float)
        Bandwidth
    
    Returns:
    K: (ndarray)
        Similarity matrix, with shape=(no. of samples, no. of samples)
    """
    N, _ = D.shape
    K = np.identity(N, dtype=np.float) # Initialise matrix
    for i in range(N):
        for j in range((i+1), N):
            sep_vec = D[i,:] - D[j,:]
            dist_sq = np.dot(sep_vec, sep_vec)
            if (dist_sq < k):
                temp = np.exp(-(1./k)*dist_sq)
                K[i,j] = temp
                K[j,i] = temp
    return K
```

### 2.2 Normalised graph Laplacian operator¶

Given the Let $T$ be the diagonal matrix of outdegrees, i.e.
\begin{equation}
T = \mathrm{diag}\Bigl(\sum*{\text{all } p} K(p,p')\Bigr)
\end{equation}
where $K*{ij} \equiv K(p*i, p\_j)$ are the components of the similarity matrix defined above. Defining the matrix $\tilde{K}$ as
\begin{equation}
\tilde{K} = T^{-1}KT^{-1},
\end{equation}
and
\begin{equation}
\tilde{T} = \mathrm{diag}\Bigl(\sum*{\text{all } p} \tilde{K}(p,p')\Bigr),
\end{equation}
the normalised graph Laplacian is then defined as
\begin{equation}
\tilde{L} = k^{-1}(I - \tilde{T}^{-1}\tilde{K})
\end{equation}
where $I$ is the $n$-dimensional identity matrix.

In [3]:

```
def constr_laplacian(K, k):
    """Construct normalised graph Laplacian matrix operator
    
    Parameters:
    K: (ndarray)
        Similarity matrix 
    k: (float)
        Bandwidth 
    
    Returns:
    L_tilde: (ndarray)
        Normalised graph Laplacian
    """
    N = K.shape[0]
    
    T = np.sum(K, axis=0)
    T_inv = np.diag(1.0/T)
    K_tilde = np.dot(np.dot(T_inv, K), T_inv)

    T_tilde = np.sum(K_tilde, axis=0)
    T_tilde_inv = np.diag(1.0/T_tilde)
    
    L_tilde = (np.identity(N) - np.dot(T_tilde_inv, K_tilde))/k

    return L_tilde
```

### 2.3 Metric Learning¶

Given a set of 2D coordinate maps $f$ and the normalised graph Laplacian $\tilde{L}$, the components of the embedded inverse metric field $h^{ij}$ is
\begin{equation}
h^{ab} = \frac{1}{2}\Bigl[\tilde{L}(f^a\cdot f^b) - f^a\cdot(\tilde{L}f^b) - f^b\cdot(\tilde{L}f^a) \Bigr],
\end{equation}
where $a,b=1,2$, and the inverse metric at point $p$ is simply $h(p)$.

In [4]:

```
def metric_learn(L_tilde, f):
    """Metric learning via the normalised Laplacian
    
    Parameters:
    L_tilde: (ndarray)
        Normalised Laplacian
    f: (ndarray)
        2D coordinates of samples (with shape=(nsamples, 2))
    
    Returns:
    h: (ndarray)
        metric, with shape=(no. of samples, 3); the three columns
        are the components h_{11}, h_{12}, and h_{22} respectively
    h_inv: (ndarray)
        inverse metric
    """
    
    N = f.shape[0] # nsamples

    # Initialise containers
    h_tilde = np.zeros(shape=(N,3))
    h = np.zeros(shape=(N,3))
    h_inv = np.zeros(shape=(N,3))

    # Define first term
    temp_1 = np.dot(L_tilde, f[:,0]*f[:,0])
    temp_2 = f[:,0]*np.dot(L_tilde, f[:,0])
    h_tilde[:,0] = 0.5*(temp_1 - 2.0*temp_2)
    
    # Define second term
    temp_1 = np.dot(L_tilde, f[:,1]*f[:,0])
    temp_2 = f[:,1]*np.dot(L_tilde, f[:,0])
    temp_3 = f[:,0]*np.dot(L_tilde, f[:,1])
    h_tilde[:,1] = 0.5*(temp_1 - temp_2 - temp_3)

    # Define third term
    temp_1 = np.dot(L_tilde, f[:,1]*f[:,1])
    temp_2 = f[:,1]*np.dot(L_tilde, f[:,1])
    h_tilde[:,2] = 0.5*(temp_1 - 2.0*temp_2)

    # Take the inverse
    for i in range(N):
        h_tmp = np.linalg.pinv(np.array([[h_tilde[i,0], h_tilde[i,1]],[h_tilde[i,1], h_tilde[i,2]]]))
        h[i,:] = -np.array([h_tmp[0,0], h_tmp[0,1], h_tmp[1,1]])

        h_inv_tmp = np.linalg.inv(h_tmp)
        h_inv[i,:] = -np.array([h_inv_tmp[0,0], h_inv_tmp[0,1], h_inv_tmp[1,1]]) 
        
    return (h, h_inv)
```

## 3. Load simulated 3D random walk data¶

The simulated motion of the particles is a random walk on the surface of an ellipsoid with axis ratios $(a,b,c) = (7,7,14)$.

The data is saved as a `csv` file, where each row represents a single observation. There are four labelled columns: 'id', representing the particle label, and 'x', 'y', 'z' the 3D coordinates. The observations within each subset of the data with the same 'id' label are ordered chronologically. Without loss of generality, every particle in this data set is made up of an equal number of observations (20).

In [5]:

```
df = pd.read_csv("./SimulationData/exampleDataPRW.csv")
```

Reformat the data into a 2D numpy array, removing the particle labels. We would like a global chart, hence the embedded 2D manifold must be homeomorphic to a 2D disc. Furthermore truncate to a subset of 150 particles, which turns out to be sufficient to learn the manifold to a high degree of accuracy:

In [6]:

```
df_new_group = df.groupby('id')
temp_df = pd.DataFrame(df_new_group['x'].max())
selected_ids = np.array(temp_df[temp_df['x'] < 0].index)[0:150]
D_temp = np.array(df.loc[df['id'].isin(selected_ids)].ix[:,'x':])
```

Finally, attach the bias point (defined when simulating the data).

In [7]:

```
# Attach the bias point
D = np.vstack((D_temp, [-7., 0., 0.]))
```

## 4. Compute global 2D chart coordinates¶

We use the Locally Linear Embedding manifold learning algorithm (https://www.cs.nyu.edu/~roweis/lle/). We set the `n_neighbors` parameter to ensure that each neighbourhood includes tracks from at least two particles.

In [8]:

```
chart_coords, _ = sklearn.manifold.locally_linear_embedding(X=D, 
                        n_neighbors=21, n_components=2, method='modified')
```

We can visualise the paths in the 2D chart

In [9]:

```
# Visualise the paths in the 2D chart
%matplotlib inline
tot_num_points = D.shape[0]
path_len = 20
num_paths = tot_num_points//path_len

for i in range(num_paths):
    x_coords = chart_coords[(i*path_len):((i+1)*path_len), 0]
    y_coords = chart_coords[(i*path_len):((i+1)*path_len), 1]
    plt.plot(x_coords, y_coords)
```

## 5. Perform metric learning¶

We begin by constructing a similarity matrix $K$. The bandwidth choice of $k=30$ is not related to the manifold learning parameter 'n\_neighbors' above. The results here can be shown to be fairly insensitive to the value of this parameter; changing k below to any value in the range $5 < k < 50$ does not affect the final result at the end.

In [10]:

```
k = 30
K = constr_sim_matrix(D, k=k) # This step should take ~1 min on a standard workstation.
```

Next we construct the normalised graph Laplacian and obtain the metric values.

In [11]:

```
L_tilde = constr_laplacian(K, k=k) # normalised Laplacian
learned_metric, learned_metric_inv = metric_learn(L_tilde, chart_coords) # metric and inverse metric
```

## 6. Find the empirical persistent angle distributions¶

Using the methods with and without the metric, we calculate the persistent angles over all steps for the particles

In [12]:

```
## Define containers
angles_euclid = np.zeros(num_paths*(path_len-2))
angles_riemann = np.zeros(num_paths*(path_len-2))

k=0
for i in range(num_paths):
    path = chart_coords[(i*path_len):((i+1)*path_len), :]
    g = learned_metric[(i*path_len):((i+1)*path_len), :] # metric
    for j in range(path_len-2):
        u = path[j+1,:]-path[j,:]
        v = path[j+2,:]-path[j+1,:]
        
        # no metric
        theta_1 = np.arccos(np.dot(u,v)/np.sqrt(np.dot(u,u)*np.dot(v,v))) 
        if (v[1] > u[1]): # Sign convention
            theta_1 = -theta_1
        angles_euclid[k] = theta_1
        
        # with metric
        gp = np.array([[g[j+1,0], g[j+1,1]], [g[j+1,1], g[j+1,2]]])
        uT = np.dot(gp,u)
        vT = np.dot(gp,v)
        theta_2 = np.arccos(np.dot(u,vT)/np.sqrt(np.dot(u,uT)*np.dot(v,vT))) 
        if (vT[1] > uT[1]): # Sign convention
            theta_2 = -theta_2
        angles_riemann[k] = theta_2
        
        k += 1
```

## 7. Compare results against the generative distribution¶

The persistent random walk angles are generated from a wrapped Normal distribution with scale parameter $\sigma=1.2$. We generate the angles by wrapping a Normal distribution 'manually':

In [13]:

```
angles_true = np.random.normal(loc=np.pi, scale=1.2, size=100*num_paths*(path_len-2)) # 100 is an arbitrary large factor

# Truncate top end
pos_ind = (angles_true >= 0)
angles_true[pos_ind] = angles_true[pos_ind] % (2*np.pi)

# Truncate bottom end
angles_true = -angles_true + 2*np.pi
pos_ind = (angles_true >= 0)
angles_true[pos_ind] = angles_true % (2*np.pi)
angles_true = -angles_true + np.pi
```

Plotting the results.

In [14]:

```
%matplotlib inline
f, ax = plt.subplots()
ax = sns.kdeplot(angles_true, color="black", clip=(-np.pi, np.pi), label="True distribution")
ax = sns.kdeplot(angles_euclid, color="r", clip=(-np.pi, np.pi), label="No metric")
ax = sns.kdeplot(angles_riemann, color="b", clip=(-np.pi, np.pi), label="With metric")
```
