# Supplementary material for: Accurate Reconstruction of Cell and Particle Tracks from 3D Live Imaging Data
Source: Cell Syst. 2016 Jul 27;3(1):102–7. doi: 10.1016/j.cels.2016.06.002 (PMC4963212; doi:10.1016/j.cels.2016.06.002)
Supplement: Data S1. This Folder Contains the Two Jupyter Notebooks for Unwrapping and Manifold Learning, Related to Experimental Procedures — Furthermore it includes the two websites for both methods. Example data are stored in the folder “SimulationData.” [file mmc6.zip › Jupyter/UnwRapping.html]

UnwRapping


In [2]:

```
library(IRdisplay)
```

# Unwrapping of Fly Data in the *R* programming language¶

### Prerequisites¶

To run the Jupyter notebook you need:

- an installation of the Jupyter notebook
- an installation of the *R* statistical environment
- and the R Kernel for the Jupyter notebook. For the latter the instructions found here are good for installing this under OSX.
- visualizing the output in *R* requires the *rgl* package, which can be installed using your *R* environment.

Provided that these packages are in place the code in this notebook should run without any further problems.

### Routines for Unwrapping Data¶

We first define a set of necessary routines

- `getAngleBias` lets us define the angle to the wound
- `getAnglePersistence` determines the angle between successive steps and hence measures the *persistence*
- `transformData`
- `unwrapData3DPlottingXY3`

In [3]:

```
getAngleBias <- function(x,y,wound){

	N = dim(x)[1]
	T = dim(x)[2]

	number = N
	steps = T
	posX = x
	posY = y
	
	# compute the angles
	alpha3 = matrix(nrow=number,ncol=steps)
	
	for (trajectory in 1:number){
		index = which(is.na(posX[trajectory,])==FALSE)
		if(length(index)>4){
			for(t in index[1:(length(index)-1)]){
				r = c(posX[trajectory,t],posY[trajectory,t]) 
				q = c(posX[trajectory,t+1],posY[trajectory,t+1]) 
				
				x = q-r
				
				temp = c(wound[1]-posX[trajectory,t],wound[2]-posY[trajectory,t])
				
				if(temp[1]>0){etha =  acos(temp[2]/sqrt(sum(temp**2)))}
				if(temp[1]<0){etha =  -acos(temp[2]/sqrt(sum(temp**2)))}
				
				x2 = c(cos(etha)*x[1]-sin(etha)*x[2],sin(etha)*x[1]+cos(etha)*x[2])
				
				if(x2[1]<0){ alpha3[trajectory,t]= -acos((temp[1]*x[1]+temp[2]*x[2])/(sqrt(sum(x**2))*sqrt(sum(temp**2))))}		
				if(x2[1]>0){ alpha3[trajectory,t]= acos((temp[1]*x[1]+temp[2]*x[2])/(sqrt(sum(x**2))*sqrt(sum(temp**2)))) }	
				if(x2[1]==0){alpha3[trajectory,t]= 0.0}
				
			}
		}
	}
	
	return(alpha3)
}


getAnglePersistence <- function(x,y){
	
	
	N = dim(x)[1]
	T = dim(x)[2]
	
	number = N
	steps = T
	
	posX = x
	posY = y

	# compute the angles
	alpha3 = matrix(nrow=number,ncol=steps)
	
	for (trajectory in 1:number){
		index = which(is.na(posX[trajectory,])==FALSE)
		if(length(index)>4){
			for(t in index[1:(length(index)-2)]){
				r = c(posX[trajectory,t],posY[trajectory,t]) 
				q = c(posX[trajectory,t+1],posY[trajectory,t+1]) 
				
				r2 = c(posX[trajectory,t+1],posY[trajectory,t+1]) 
				q2 = c(posX[trajectory,t+2],posY[trajectory,t+2]) 
				
				x = q-r
				
				temp = c(1,0)
				temp = q2-r2
				
				if(temp[1]>0){etha =  acos(temp[2]/sqrt(sum(temp**2)))}
				if(temp[1]<0){etha =  -acos(temp[2]/sqrt(sum(temp**2)))}
				
				x2 = c(cos(etha)*x[1]-sin(etha)*x[2],sin(etha)*x[1]+cos(etha)*x[2])
				
				if(x2[1]<0){ alpha3[trajectory,t]= -acos((temp[1]*x[1]+temp[2]*x[2])/(sqrt(sum(x**2))*sqrt(sum(temp**2))))}		
				if(x2[1]>0){ alpha3[trajectory,t]= acos((temp[1]*x[1]+temp[2]*x[2])/(sqrt(sum(x**2))*sqrt(sum(temp**2)))) }	
				if(x2[1]==0){alpha3[trajectory,t]= 0.0}
				
			}
		}
	}
	
	return(alpha3)
}


transformData <- function(Data,cx,cy,cz){
	
	shift = c(0,0,cz[1])
	
	Data[[1]] = Data[[1]]-shift[1]
	Data[[2]] = Data[[2]]-shift[2]
	Data[[3]] = Data[[3]]-shift[3]
		
	return(Data)
	

}


unwrapData <- function(Data,cx,cy,cz){
	

	#slice: to flatten yz-curve
	Ns = 10
	
	myColors = colors()[c(404,38,104,105,8,400,600,625)]
	
	x = Data[[1]]
	y = Data[[2]]
	z = Data[[3]]
	
	
	xt = matrix(NA,dim(x)[1],dim(x)[2])
	yt = matrix(NA,dim(x)[1],dim(x)[2])
	zt = matrix(NA,dim(x)[1],dim(x)[2])
	
	limX = seq((cx[1]-1),(cx[2]+1),by=(cx[2]-cx[1]+2)/Ns)	
	
	for(k in 1:Ns){
		temp = (x>limX[k])&(x<limX[k+1])
		
		
		# find circle
		
		datX = x[temp]
		datY = y[temp]
		datZ = z[temp]
		
		dat = cbind(datX,datY,datZ)
		
		ellipse <-function(p)	{
			sum((p[3]*p[2]-sqrt(p[3]**2*dat[,2]**2+p[2]**2*(dat[,3]-p[1])**2))**2)
		}
		
		minimise <- optim(par=c(2*max(dat[,2]),2*max(dat[,2]),2*max(dat[,2])),fn=ellipse,method="L-BFGS-B",lower=c(0,abs(min(dat[,2])-max(dat[,2]))/3,abs(min(dat[,3])-max(dat[,3]))/3),upper=c(5*max(dat[,3]),5*max(dat[,2]),5*max(dat[,3])))
		param = minimise$par
		
		
		if(k==1){
			plot3d(datX,datY,datZ,col=rainbow(Ns)[k],box=FALSE,xlab="x",ylab="y",zlab="z",xlim=c(-20,20),ylim=c(-20,20),zlim=c(0,35))
		}
		else{
			plot3d(datX,datY,datZ,col=rainbow(Ns)[k],add=TRUE)
		}
		
		
		ytemp = y
		ztemp = z
		dist = matrix(NA,dim(y)[1],dim(y)[2])
		maxD = rep(NA,dim(temp)[1])
		minD = rep(NA,dim(temp)[1])
		
		for(i in 1:dim(temp)[1]){
			ind = which(temp[i,]==TRUE)
			if(length(ind)>0){
				v1 = cbind(y[i,ind],z[i,ind]-param[1])
				lengthV1 = sqrt(v1[,1]**2+v1[,2]**2)
				
				t_hat = atan(param[2]/param[3]*(z[i,ind]-param[1])/y[i,ind])
				v_hat = cbind(param[2]*cos(t_hat),param[3]*sin(t_hat))
				lengthV_hat = sqrt(v_hat[,1]**2+v_hat[,2]**2)
				
				d_hat = lengthV_hat-lengthV1
				
				# arc length
				integrand <- function(t,a,b){
					result = sqrt(a**2*sin(t)**2+b**2*cos(t**2))
					return(result)
				}
				
				s = rep(NA,length(ind))
				for(j in 1:length(ind)){
					s[j] = integrate(integrand, lower=-pi/2, upper=t_hat[j],param[2],param[3])[[1]]
				}
				
				yt[i,ind] = s #y[i,ind]
				xt[i,ind] = x[i,ind]  
				zt[i,ind] = -param[2]+d_hat
				
				halfLength = integrate(integrand, lower=-pi/2, upper=pi/2,param[2],param[3])[[1]]
				
				sign = which(y[i,ind]<0)
				yt[i,ind[sign]] = yt[i,ind[sign]]-halfLength
				
			}
			
		}

		plot3d(xt,yt,zt+30,add=TRUE,col=rainbow(Ns)[k])
		
	}	
	
	COMPUTE=TRUE
	if(COMPUTE==TRUE){
		
		#slice: to flatten xz-curve
		Ns = 1
				
		myColors = colors()[c(404,38,104,105,8,400,600,625)]
		
		# change of variables
		x = yt
		y = xt
		z = zt
		
		xt = matrix(NA,dim(x)[1],dim(x)[2])
		yt = matrix(NA,dim(x)[1],dim(x)[2])
		zt = matrix(NA,dim(x)[1],dim(x)[2])
		
		cx = c(min(x,na.rm=TRUE),max(x,na.rm=TRUE))
		
		#shift all to positive values in z
		z = z + abs(min(z,na.rm=TRUE))

		limX = seq((cx[1]-1),(cx[2]+1),by=(cx[2]-cx[1]+2)/Ns)
				
		for(k in 1:Ns){
			temp = (x>limX[k])&(x<limX[k+1])
			
			# find ellipse
			datX = x[temp]
			datY = y[temp]
			datZ = z[temp]
			
			ind = which(is.na(datX)==TRUE)
			dat = cbind(datX,datY,datZ)
			
			
			ellipse <-function(p)	{
				sum((p[3]*p[2]-sqrt(p[3]**2*dat[,2]**2+p[2]**2*(dat[,3]-p[1])**2))**2)
			}
			
			minimise <- optim(par=c(2*max(dat[,2]),2*max(dat[,2]),2*max(dat[,2])),fn=ellipse,method="L-BFGS-B",lower=c(0,abs(min(dat[,2])-max(dat[,2]))/3,abs(min(dat[,3])-max(dat[,3]))/3),upper=c(5*max(dat[,3]),5*max(dat[,2]),5*max(dat[,3])))
			
			param = minimise$par
			print(param)
			

			ytemp = y
			ztemp = z
			dist = matrix(NA,dim(y)[1],dim(y)[2])
			maxD = rep(NA,dim(temp)[1])
			minD = rep(NA,dim(temp)[1])
			
			for(i in 1:dim(temp)[1]){
				
				ind = which(temp[i,]==TRUE)
				v1 = cbind(y[i,ind],z[i,ind]-param[1])
				lengthV1 = sqrt(v1[,1]**2+v1[,2]**2)
				
				t_hat = atan(param[2]/param[3]*(z[i,ind]-param[1])/y[i,ind])
				v_hat = cbind(param[2]*cos(t_hat),param[3]*sin(t_hat))
				lengthV_hat = sqrt(v_hat[,1]**2+v_hat[,2]**2)

				d_hat = lengthV_hat-lengthV1
				
				# arc length
				integrand <- function(t,a,b){
					result = sqrt(a**2*sin(t)**2+b**2*cos(t**2))
					return(result)
				}

				s = rep(NA,length(ind))
				for(j in 1:length(ind)){
					s[j] = integrate(integrand, lower=-pi/2, upper=t_hat[j],param[2],param[3])[[1]]
				}
				
				# change back variables when saving as xt, yt, zt
				yt[i,ind] = x[i,ind]
				xt[i,ind] = s  #y[i,ind]
				zt[i,ind] = d_hat

				halfLength = integrate(integrand, lower=-pi/2, upper=pi/2,param[2],param[3])[[1]]
				
				sign = which(y[i,ind]<0)
				xt[i,ind[sign]] = xt[i,ind[sign]]-halfLength
		
			}

		}	
		
	}
	
	plot3d(xt,yt,zt+33,add=TRUE,col="grey")

	
	Data[[1]]=xt
	Data[[2]]=yt
	Data[[3]]=zt
	
	return(Data)
	
}

formatData <- function(data){
	
	ids = data[,1]
	idUnique = unique(ids)
	
	
	ind = which(ids==idUnique[1])
	x = matrix(data[ind,2],1,length(ind))
	y = matrix(data[ind,3],1,length(ind))
	z = matrix(data[ind,4],1,length(ind))
	
	for(i in 2:length(idUnique)){
		
		
		ind = which(ids==idUnique[i])
		
		if(length(ind)==dim(x)[2]){
			x = rbind(x,data[ind,2])
			y = rbind(y,data[ind,3])
			z = rbind(z,data[ind,4])
		}
		
		
		if(length(ind)<dim(x)[2]){
			n = dim(x)[2]-length(ind)
			temp = c(data[ind,2],rep(NA,n))
			x = rbind(x,temp)
			
			temp = c(data[ind,3],rep(NA,n))
			y = rbind(y,temp)
			
			temp = c(data[ind,4],rep(NA,n))
			z = rbind(z,temp)
		}
		
		if(length(ind)>dim(x)[2]){
			n = -dim(x)[2]+length(ind)
			temp1 = matrix(NA,dim(x)[1],n)
			temp2 = cbind(x,temp1)
			x = rbind(temp2,data[ind,2])
			
			temp2 = cbind(y,temp1)
			y = rbind(temp2,data[ind,3])
			
			temp2 = cbind(z,temp1)
			z = rbind(temp2,data[ind,4])
		}
		
		
		
	}
	
	Data = list()
	Data[[1]] = x
	Data[[2]] = y
	Data[[3]] = z
	
	return(Data)
	
}
```

### Graphics Requirements¶

The `rgl` library is required to visualize the output in the correct 3D setting

In [4]:

```
library(rgl)
```

## Unwrapping Fly Embryo Data¶

Here we provide an example data set (exampleDataBPRW.txt) in the folder 'SimulatedData'.

This data set describes simulated cell trajectories based on a biased persistent random walk on an ellipsoid surface. Each 3 columns are the x-, y- and z- coordinates of a cell track over time.

The target of the biased cells (wound) is at position (-7, 0, 0), which needs to be defined in the first step.

Then we read in the data and bring it into the correct format.

In [5]:

```
wound = c(-7,0,0)

	
###########################################
# 1) read in the data and format them
###########################################


# the data are saved as csv file, which can be opened with any text editor or excel. 
# The first column is the cell trajectory identifier.
# The second column is the x coordinate
# The third column is the y coordinate
# The fourth column is the z coordinate


dat = read.csv(file="./simulatedData/exampleDataPRW.csv")

Data = formatData(dat)

N = dim(Data[[1]])[1]
```

We are now in a position to analyze the data.

### Plot Data in 3D¶

We begin by plotting the trajectory data in 3D (using *rgl*).

This allows us to get an idea of the geometry of the data. We can then clearly see that the trajectory data lie on a surface of an ellipsoid with radii 6, 6 and 18.

In [8]:

```
###########################################
# 2) plot data in 3D
###########################################
par3d("windowRect"= c(0,0,1200,1200))
plot3d(Data[[1]],Data[[2]],Data[[3]],viewport=c(0,0,1000,500),box=FALSE,type="p",lwd=1,cex=0.4,col="grey",aspect=c(14,7,7),xlab="x",ylab="y",zlab="z")
for(i in 1:N){
	plot3d(Data[[1]][i,],Data[[2]][i,],Data[[3]][i,],box=FALSE,type="l",lwd=2,col=i,add=TRUE)
}
rgl.postscript("dataplots.pdf","pdf")
rgl.snapshot("dataplots.png")
display_png(file='dataplots.png')
```

### Analysis of random walk statistics in the 2D projection¶

We then calculate the bias and persistence of the random walk data in the conventional projection down to 2D.

In [6]:

```
###########################################
# 3) compute statistics in xy-projection
###########################################

x = Data[[1]]
y = Data[[2]]
z = Data[[2]]

alphaXY = getAngleBias(x,y,w=c(-7,0))
betaXY = getAnglePersistence(x,y)
```

### Unwrapping of the data onto a flat manifold¶

We next transform the data onto a flat space using the unwrapping method by calling the routine unwrapData(). If a simple representation of the manifold is available (such as a cylinder or ellipsoid) then we can unwrap the data by mapping the correct positions on the manifold (akin to cartographic projections) in a way that maintains the angles correctly. The routine will create a 3D graphical presentation of the progress of the unwrapping procedure. Firstly, the original data are plotted in 3D and then shifted to be suitable for the unwrapping using the routine transformData(). The original data are then clustered along the x-axis and plotted in 3D, where each cluster is shown in a different colour. Next, an ellipse is fitted to each cluster. The data are then unrolled onto a new space based on the characteristics of the fitted ellipse (for exact details see section 4). This first step is an approximation to manifold learning techniques. In the following the data obtained from the first step are further unrolled by fitting an ellipse onto a flat space. The routine will plot the transformed data in grey.

Here this is done for the simulated data on an ellipsoid.

In [7]:

```
###########################################
# 5) unwrap data
###########################################

cx = c(min(Data[[1]],na.rm=TRUE),max(Data[[1]],na.rm=TRUE))
cy = c(min(Data[[2]],na.rm=TRUE),max(Data[[2]],na.rm=TRUE))
cz = c(min(Data[[3]],na.rm=TRUE),max(Data[[3]],na.rm=TRUE))

DataT = transformData(Data,cx,cy,cz)

par3d("windowRect"= c(0,0,1200,1200))
plot3d(Data[[1]],Data[[2]],Data[[3]],box=FALSE,type="p",lwd=1,cex=0.4,col="grey",aspect=c(14,7,7))
for(i in 1:N){
	plot3d(DataT[[1]][i,],DataT[[2]][i,],DataT[[3]][i,],box=FALSE,type="l",lwd=2,col=i,add=TRUE)
}

cx = c(min(DataT[[1]],na.rm=TRUE),max(DataT[[1]],na.rm=TRUE))
cy = c(min(DataT[[2]],na.rm=TRUE),max(DataT[[2]],na.rm=TRUE))
cz = c(min(DataT[[3]],na.rm=TRUE),max(DataT[[3]],na.rm=TRUE))

DataU = unwrapData(DataT,cx,cy,cz)

xt = DataU[[1]]
yt = DataU[[2]]
zt = DataU[[3]]

rgl.snapshot("dataplots1.png")
display_png(file='dataplots1.png')
```

```
[1]  7.120469 18.021074  6.150133
```

### Analysis of random walk statistics in the manifold projection¶

We can now calculate the bias and persistence of the random walk data in the 2D manifold projection. Again, this is done via the routines `getAngleBias()` and `getAnglePersistence()` by passing the relevant coordinates.

In [8]:

```
###########################################
# 6) compute stats for unwrapped data
###########################################


# extract the position of the unwrapped wound
wound = c(xt[dim(xt)[1],1],yt[dim(yt)[1],1],zt[dim(zt)[1],1])

alphaUnwr = getAngleBias(xt,yt,w=wound)
betaUnwr = getAnglePersistence(xt,yt)
```

### Comparison of inferred biased and persistence behaviour in the 2D *x-y* and unwrapped (manifold) projection¶

Finally we can compare the computed statistics for the *xy*-projection with the statistics computed from the unwrapped data. This is for example done via plotting the histograms and densities for the bias and persistent distributions. Doing so, we observe strong artefacts in the obtained distributions based on the *xy*-projection. By contrast, unwrapping manages to recover the expected bias and persistence distributions.

In [10]:

```
###########################################
# 7) Plot results
###########################################

layout(matrix(1:4,2,2,byrow=TRUE))

hist(alphaXY,main="bias distribution: xy-proj.",xlab="alpha",breaks=30,freq=FALSE)
d=density(alphaXY,from=-pi,to=pi,na.rm=TRUE,adjust=2)
points(d[[1]],d[[2]],type="l",col="red",lwd=2)

hist(betaXY,main="persistence distribution: xy-proj.",xlab="beta",breaks=30,freq=FALSE)
d=density(betaXY,from=-pi,to=pi,na.rm=TRUE,adjust=2)
points(d[[1]],d[[2]],type="l",col="red",lwd=2)


hist(alphaUnwr,main="bias distribution: unwrapping",xlab="alpha",breaks=30,freq=FALSE)
d=density(alphaUnwr,from=-pi,to=pi,na.rm=TRUE,adjust=2)
points(d[[1]],d[[2]],type="l",col="red",lwd=2)

hist(betaUnwr,main="persistence distribution: unwrapping",xlab="beta",breaks=30,freq=FALSE)
d=density(betaUnwr,from=-pi,to=pi,na.rm=TRUE,adjust=2)
points(d[[1]],d[[2]],type="l",col="red",lwd=2)
```

xml version="1.0" encoding="UTF-8"?

In [ ]:

```

```
